# Surface engineering *Salmonella* with pH‐responsive polyserotonin and self‐activated DNAzyme for better microbial therapy of tumor

**DOI:** 10.1002/EXP.20230017

**Published:** 2023-10-05

**Authors:** Lina Guo, Hao Chen, Jinsong Ding, Pengfei Rong, Ming Sun, Wenhu Zhou

**Affiliations:** ^1^ Xiangya School of Pharmaceutical Sciences Central South University Changsha China; ^2^ Department of Pathology Shihezi University School of Medicine Shihezi China; ^3^ Department of Radiology The Third Xiangya Hospital Central South University Changsha China; ^4^ Division of Systems Pharmacology and Pharmacy Leiden Academic Center for Drug Research Leiden University Leiden The Netherlands

**Keywords:** bacteria‐mediated cancer therapy, immunotherapy, self‐polymerization, surface coating, targeting

## Abstract

Bacteria‐based microbial immunotherapy shows various unique properties for tumor therapy owing to their active tropism to tumor and multiple anti‐tumor mechanisms. However, its clinical benefit is far from satisfactory, which is limited by rapid systemic clearance and neutrophils‐mediated immune restriction to compromise the efficacy, as well as non‐specific distribution to cause toxicity. To address all these limitations, herein we reported a polyserotonin (PST) coated *Salmonella* (*Sal*) with surface adsorption of DNAzyme (Dz)‐functionalized MnO_2_ nanoparticles (DzMN) for tumor therapy. PST could facilely coat on *Sal* surface via oxidation and self‐polymerization of its serotonin monomer, which enabled surface stealth to avoid rapid systemic clearance while maintaining the tumor homing effect. Upon targeting to tumor, the PST was degraded and exfoliated in response to acidic tumor microenvironment, thus liberating *Sal* to recover its anti‐tumor activities. Meanwhile, the DzMN was also delivered into tumor via hitchhiking *Sal*, which could release Dz and Mn^2+^ after tumor cells internalization. The Dz was then activated by its cofactor of Mn^2+^ to cleave target PD‐L1 mRNA, thus serving as a self‐activated system for gene silencing. Combining *Sal* and Dz for immune activation and PD‐L1 knockdown, respectively, anti‐tumor immunotherapy was achieved with enhanced efficacy. Notably, PST coating could significantly decrease infection potential and non‐specific colonization of *Sal* at normal organs, achieving high in vivo biosafety. This work addresses the key limitations of *Sal* for in vivo application via biomaterials modification, and provides a promising platform for better microbial immunotherapy.

## Graphical abstract

This study presents a novel surface modification approach aimed at enhancing the biomedical applications of *Salmonella*. By employing a facile surface engineering technique involving pH‐responsive polyserotonin (PST) and self‐activated DNAzyme, the immune clearance and non‐specific colonization of *Salmonella* can be effectively mitigated. The PST coating exhibits the ability to disassemble in acidic tumor microenvironments, thereby facilitating the controlled release of *Salmonella* and DNAzyme, leading to improved therapeutic outcomes.

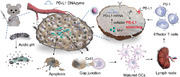

## 1 INTRODUCTION

Cancer is one of the major diseases that endanger human health and cause high mortality worldwide. Over the past decades, unremitting efforts have been made to combat cancer. Besides surgical intervention, chemotherapy and radiotherapy are the most commonly treatment strategies, while their therapeutic outcomes are restricted by the severe side‐effects and low treatment efficacy.^[^ ^1^ ^]^ To this end, recent years have witnessed a surge in the development of immunotherapy that aims at activating the immune systems of host to attack tumor cells. Immunotherapy is regarded as the most promising strategy to control tumor, which has revolutionized the cancer therapy to provide new hope for tumor patients. The current immunotherapy used in the clinic includes immune checkpoint blockade therapy and chimeric antigen receptor T cell therapy.^[^ ^2^ ^]^ Compared to the conventional cancer therapies, immunotherapy can effectively suppress tumor metastasis and recurrence with long‐term immune memory.^[^ ^3^ ^]^ However, its response rate is still unsatisfactory, and the side‐effects such as immune‐related toxicities usually occur due to the lack of suitable drug delivery systems.^[^ ^4^ ^]^ Therefore, the development of better immunotherapy with improved efficacy and lower side‐effects is highly desired in clinical application.

Actually, bacterial therapy is the earliest attempt of tumor immunotherapy, which can date back to 1868 when *Streptococcus pyogenes* was used to treat sarcoma patients.^[^ ^5^ ^]^ The clinical efficacy of bacteria‐based immunotherapy has been demonstrated a hundred years ago, although the detailed mechanisms were elusive at that time. Along with the blooming of cancer immunotherapy, researchers began to rethink the use of bacteria for tumor therapy, and found that bacteria could exert their anti‐tumor effect via various mechanisms,^[^ ^5^, ^6^ ^]^ in which the activation of anti‐tumor immunity is the most critical one.^[^ ^7^ ^]^ Compared to other types of therapeutic modalities, bacteria‐based microbial immunotherapy showed various intrinsic advantages. For example, the anaerobic and facultative anaerobes with active tropism can colonize at tumor site by virtue of the hypoxia nature of tumor,^[^ ^8^ ^]^ and can perform persistent anti‐tumor effect at tumor site via self‐reproduction.^[^ ^9^ ^]^ In this regard, bacteria are self‐drug delivery systems with tumor targeting capability for amplified tumor immunotherapy.

Among various bacteria, *Salmonella* (*Sal*) stands out because of its excellent tumor targeting, facile genetic engineering and significant anti‐tumor effects,^[^ ^10^ ^]^ and the engineered *Sal* strain VNP20009 with purI and msbB gene deletions has entered phase I clinical trial.^[^ ^11^, ^12^ ^]^ Unfortunately, the results of clinical experiments were somehow frustrated, in which no significant anti‐tumor effects were observed while dose‐dependent toxicity occurred.^[^ ^11^, ^12^ ^]^ Further studies showed that the poor treatment outcome can be ascribed to the rapid systemic clearance of bacteria by anti‐infection immunity of host body, resulting in the lack of bacterial colonization in tumor.^[^ ^13^, ^14^ ^]^ Even after colonizing into tumor, the host neutrophils are also recruited by *Sal* to strictly constrain bacteria in the central necrotic area of tumor, which also compromises the anti‐tumor efficacy.^[^ ^15^, ^16^ ^]^ Collectively, the rapid system clearance and immune restriction are the two major barriers to limit the therapeutic benefits of *Sal*. In addition, non‐specific distribution and proliferation of bacteria in normal organs cause the dose‐dependent toxicity.^[^ ^17^ ^]^ Therefore, rational engineering is still required to protect *Sal* during blood circulation and enhance its efficacy at tumor site, but detoxify at normal tissues to minimize side‐effects.

To detoxify bacteria, various surface decoration strategies have been explored.^[^ ^18^, ^19^, ^20^ ^]^ For example, bacteria camouflaged with erythrocyte membranes showed lower inflammatory reaction and clearance, which was benefited from stealthy effect and anti‐phagocytic nature of erythrocyte membranes.^[^ ^21^, ^22^ ^]^ The biomaterials such as polydopamine (PDA) were also employed to coat on bacteria surface for detoxification.^[^ ^23^, ^24^ ^]^ Compared with cell membrane, PDA coating was advantageous for extra drug loading capacity and its intrinsic photothermal effects to achieve combinatorial therapy.^[^ ^23^, ^24^, ^25^, ^26^, ^27^, ^28^, ^29^ ^]^ However, accompanied by the surface modification for detoxification, the anti‐tumor activity of bacteria is also alleviated since the immune stimulation effect is highly dependent on the immunogenicity of bacteria surface.^[^ ^7^ ^]^ Ideally, the decoration should be stably attached on bacteria surface during circulation and biodistribution to avoid rapid clearance and toxicity, while could be exfoliated from bacteria surface upon reaching the tumor tissue to recover anti‐tumor activity. However, no such decoration strategy has been reported yet.

**SCHEME 1:**
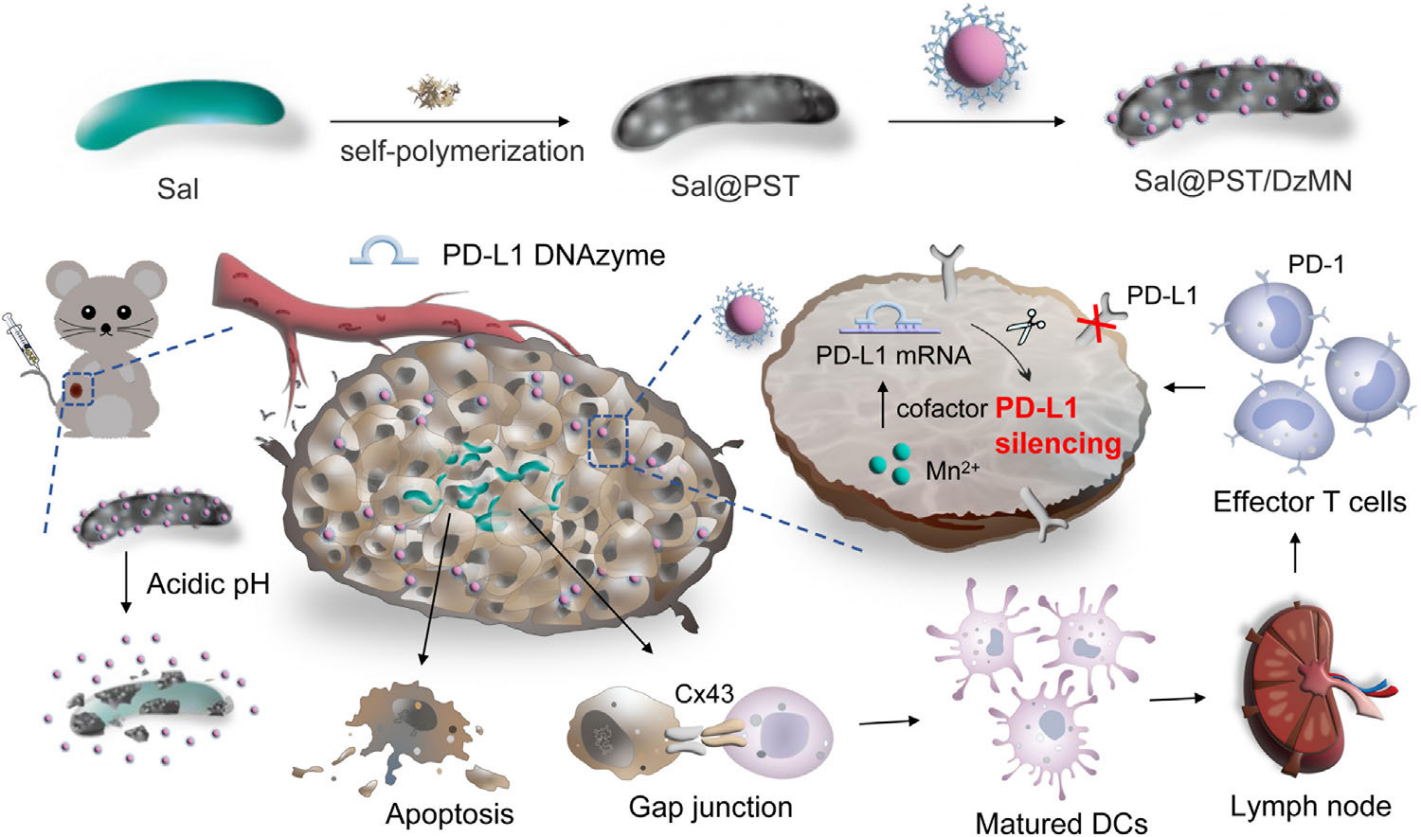
Schematic illustrating the construction of (A) *Sal*@PST/DzMN, and (B) its function mechanisms in tumor tissue for enhanced immunotherapy.

Interestingly, we recently reported a versatile coating polymer called polyserotonin (PST), which could facilely decorate on virtually any bulk or nanoparticles surfaces with properties quite similar to PDA.^[^ ^30^ ^]^ Just like PDA formed by oxidation and self‐polymerization of dopamine,^[^ ^31^, ^32^ ^]^ PST was synthesized via oxidization and self‐polymerization of its serotonin monomer. The PST possessed comparable biochemical properties to PDA but lower cytotoxicity and reduced protein corona formation.^[^ ^33^ ^]^ Notably, PST is a reversible coating that could degrade in acidic tumor microenvironment. We therefore reason that PST is a promising coating material to promote the biomedical applications of *Sal*. In this work, we fabricated and systematically characterized PST coated *Sal*, and the resulting *Sal*@PST was simply prepared with significant detoxification (Scheme 1). Importantly, such PST coating could be exfoliated from *Sal* surface at tumor site in a pH‐dependent manner to restore anti‐tumor activities of *Sal*. To further enhance the anti‐tumor immunity, a DNAzyme (Dz) conjugated MnO_2_ nanoparticle (DzMN) was prepared and adsorbed on the surface of *Sal*@PST via interfacial interaction between DNA and PST, forming a hybrid structure of *Sal*@PST/DzMN. With tumor homing effect of *Sal* as hitchhiking delivery, *Sal*@PST/DzMN could effectively accumulate into tumor, and release its components by virtue of PST degradation to exert their respective functions. Specifically, the liberated *Sal* colonized in necrotic tumor regions, could directly damage tumor cells to induce apoptosis and promote antigen presentation via the formation of gap‐junction between tumor cells and dendritic cells (DCs). Meanwhile, DzMN was internalized by tumor cells at peripheral region, and collapsed to release Mn^2+^ and Dz. Dz used in this work is a type of DNA‐based catalyst, which could cleave any target mRNA of interest in the presence of metal cofactors such as Zn^2+^, Ca^2+^, and Mn^2+^.^[^ ^34^, ^35^, ^36^ ^]^ Therefore, the co‐release of Mn^2+^ and Dz served as a self‐activated system for effective gene knockdown.^[^ ^37^, ^38^, ^39^, ^40^ ^]^ Upon Dz activation, the target PD‐L1 was silenced to enhance immune activation by blocking the immune checkpoint, leading to an augmented anti‐tumor immunotherapy.

## 2 RESULTS AND DISCUSSIONS

### 2.1 Self‐polymerization of 5‐HT to realize PST coating on *Sal* surface

**FIGURE 1:**
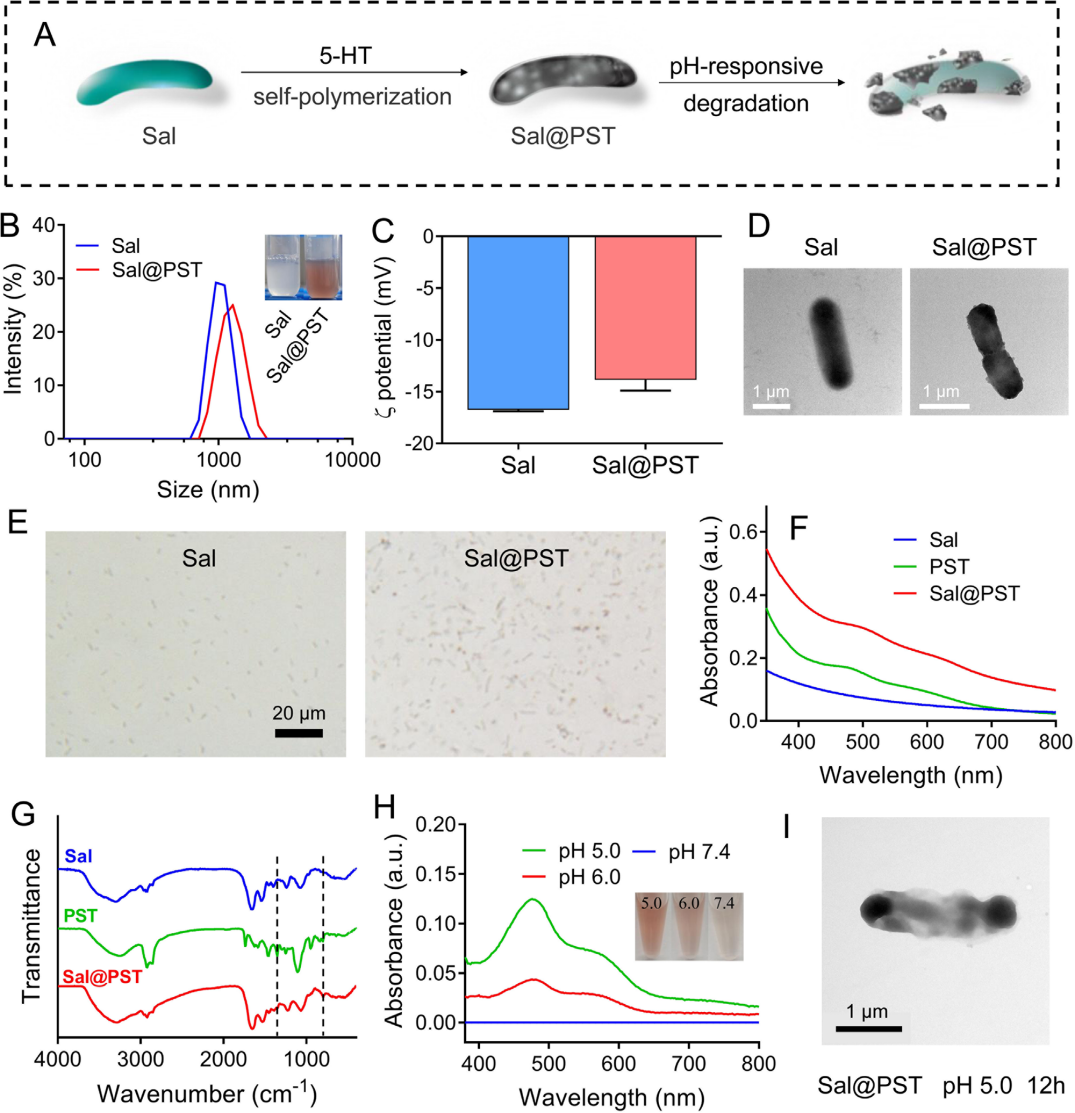
Preparation and characterizations of *Sal*@PST. (A) Schematic illustration of preparing *Sal*@PST with pH‐responsive degradation of the polyserotonin (PST) coating. (B) The size distribution and appearance, (C) ζ potential (*n* = 3), (D) Transmission electron microscopy (TEM) images and (E) micrograph of *Salmonella (* *Sal)* and *Sal*@PST. Scale bar = 20 μm. (F) UV–vis spectrum and (G) Fourier transform infrared spectrum of *Sal*, PST, and *Sal*@PST. The black dashed lines indicated the characteristic peaks of PST. (H) The absorbance and appearance of the supernatant after incubating *Sal*@PST in buffers with various pH (7.4/6.0/5.0). (I) The TEM image of *Sal*@PST after incubating in buffer (pH 5.0) for 12 h. Scale bar = 1 μm. Data were presented as mean ± SD.

According to our previous report,^[^ ^30^ ^]^ the surface PST coating was realized via oxidation and self‐polymerization of serotonin (5‐HT) monomer under alkaline conditions (Figure 1A). The resulting *Sal*@PST exhibited a slight increase of particle size from 1040 to 1241 nm and color change from white to brownish red (Figure 1B). The ζ potential of *Sal* was −16.7 mV (Figure 1C), which could be ascribed to the multiple negative biomacromolecules on their surface, such as lipopolysaccharide and lipoprotein.^[^ ^41^ ^]^ Upon PST coating, the surface became less negative owing to the abundant amino groups in polymer structure.^[^ ^33^ ^]^ From the transmission electron microscopy (TEM) images (Figure 1D), the *Sal*@PST maintained the rodlike shape of the pristine *Sal*, while the PST shell was noticed on the surface. The morphology of bacteria can also be directly observed using optical microscope (Figure 1E), which clearly showed the increase of particle size after PST coating. While the shape was unchanged from optical microscope, the contrast was significantly enhanced owing to strong UV–vis absorbance of PST. Indeed, *Sal*@PST exhibited specific absorbance peaks at 500–600 nm in the UV–vis spectrum (Figure 1F), confirming the successful PST coating. In addition, Fourier transform infrared (FT‐IR) spectrum of *Sal*@PST was also measured (Figure 1G), which exhibited several characteristic peaks assigned to *Sal* or PST polymer, respectively. More specifically, the FT‐IR spectrum of *Sal*@PST displayed characteristic peaks at 1350 and 800 cm^−1^, which can be attributed to the stretching vibration of the C─N bond and the out‐of‐plane bending vibration of the N─H bond, respectively. These peaks correspond to the presence of the nitrogen‐containing heterocyclic structure in PST, confirming its successful coating onto the surface of *Sal*. All these results confirmed the successful coating of PST on *Sal* surface.

A notable property of such PST coating is its reversible exfoliation owing to PST degradation at acidic pH (Figure 1A).^[^ ^30^ ^]^ To confirm this, *Sal*@PST was treated with buffers with different pHs for 12 h, and the supernatant was collected for characterization (Figure 1H). The color of the supernatant became darkened and the absorbance was increased along with pH decrease, suggesting more PST degradation under acidic conditions. This result was highly consistent with our previous work,^[^ ^30^ ^]^ in which PST could be degraded at pH 6 or lower, and the degradation was accelerated with lower pH. Importantly, the supernatant merely showed any UV–vis absorbance with clear color at pH 7.4. Therefore, the PST could be stably coated on the *Sal* surface under physiological pH while rapidly exfoliated from *Sal* after being delivered into acidic tumor microenvironment. This is critically important for biosafe blood circulation to minimize potential toxicity and effective recovery of anti‐tumor activity within acidic tumor microenvironment. After acidic treatment, the morphology of *Sal*@PST was further observed by TEM, which showed a rough surface with PST shell exfoliation (Figure 1I).

### 2.2 Detoxification of *Sal* upon PST coating and its pH‐responsive exfoliation to restore anti‐tumor activities

**FIGURE 2:**
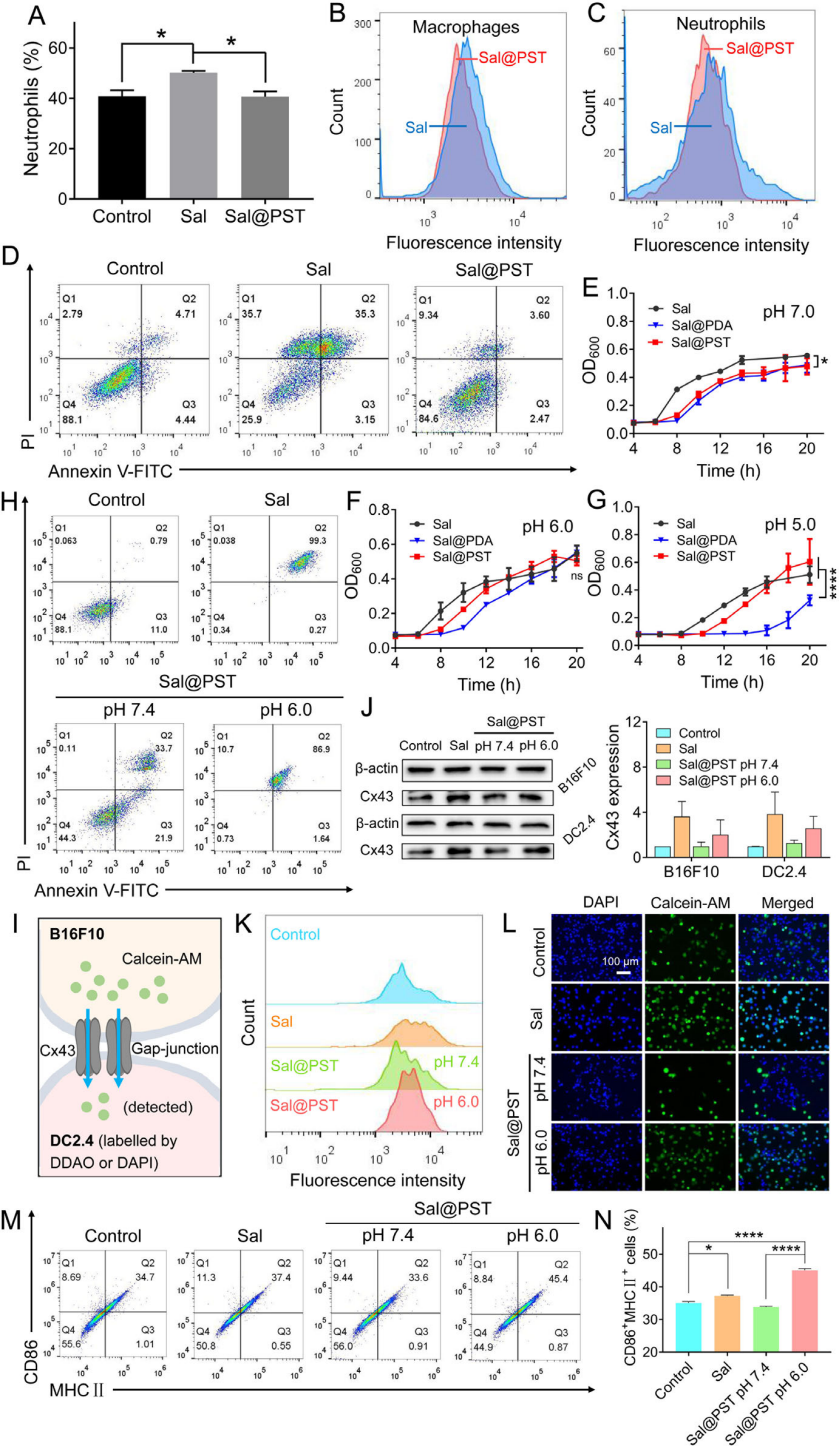
Assessment of bioactivities of *Sal*@PST. (A) The ratio of neutrophils in the blood of mice after different treatments (*n* = 3). The flow cytometry to probe phagocytosis of *Salmonella* (*Sal*) by (B) macrophages and (C) neutrophils. (D) Apoptosis analysis of HEK293T cells after different treatments. The growth curves of *Sal*, *Sal*@PDA, and *Sal*@PST in LB broth at (E) pH 7.0, (F) pH 6.0, and (G) pH 5.0 (*n* = 3). (H) Apoptosis analysis of B16F10 cells after different treatments. (I) Schematic illustrating the formation of gap‐junction via Cx43 protein to allow dye transfer. The B16F10 and DC2.4 cells were labelled with gap‐junction diffusible dye calcein‐AM and nontransferable dye DDAO or DAPI, respectively. After co‐incubation, the fluorescence signals of calcein‐AM in DC2.4 cells were detected. (J) The protein expression of Cx43 in B16F10 and DC2.4 cells after different treatments detected by WB (*n* = 3). (K) The flow cytometry results and (L) fluorescence images of calcein‐AM transferred from B16F10 cells to DC2.4 cells, as evidenced by the increased fluorescence intensity and emergence of fluorescence colocalization. Scale bar = 100 μm. (M) The flow cytometry results and (N) quantification of the expression of antigen‐presenting molecules MHC II and costimulatory molecules CD86 in DC2.4 cells post different treatments (*n* = 3). Data were presented as mean ± SD. Statistical comparisons were performed using one‐way ANOVA for (A), (E–G), and (N). ns, not significant, *p < 0.05, ****p < 0.0001.

As a foreign microorganism, *Sal* could be recognized and eliminated by specific immune cells such as neutrophils and macrophages after being intravenously injected into the bloodstream,^[^ ^13^, ^14^ ^]^ which presents the first biological barrier for tumor colonization of *Sal* and its anti‐tumor efficacy. Since the immune recognition is achieved based on the pathogen‐associated molecular patterns (PAMPs) on *Sal* surface,^[^ ^42^ ^]^ we aimed to employ PST to shield *Sal* surface, which may decrease the recognition and immune clearance. To examine this, we first injected *Sal* or *Sal*@PST in mice, and the ratio of neutrophils in the blood was measured (Figure 2A). Upon treatment with *Sal*, the number of neutrophils significantly increased since they are the most important innate immune cells to combat the invaded pathogens. For the *Sal*@PST group, by contrast, we did not observe any change of neutrophils number, suggesting the immunogenicity decrease of *Sal* upon PST coating during circulation. We then directly studied the recognition and phagocytosis of *Sal* or *Sal*@PST by the immune cells (Figure 2B,C and Figure S1). From the intensity of the flow cytometry, it is clearly seen that, compared to that of *Sal*, the engulfment of *Sal*@PST by macrophages and neutrophils was strongly weakened, confirming the surface stealthy effect of PST coating to decrease immune cells opsonization.

Besides rapid clearance, the other critical limitation related to biological applications of *Sal* is the off‐target biodistribution to cause severe side‐effects. For example, *Sal* could directly induce cell apoptosis or compete with the cells for nutrition to induce cell death.^[^ ^43^ ^]^ In this aspect, *Sal*@PST may hold another advantage over *Sal* owing to surface PST for detoxification. To demonstrate this, the normal HEK293T cells were chosen as proof‐of‐concept, and the cytotoxicity was evaluated by Annexin V‐FITC and PI double staining (Figure 2D and Figure S2). After 24 h incubation, only ∼25% live cells were observed with *Sal* treatment, demonstrating that the bacteria were highly toxic to cells when co‐incubation. For *Sal*@PST group, by contrast, over 80% of cells were still alive. Therefore, PST coating could significantly detoxify *Sal* to improve the safety. One underling reason may be the stealthy effect of PST to decrease the immunogenicity as described above, and the other reason may be attributable to the growth restriction of PST shell, which abolishes the nutrition competition. To confirm this, we investigated the bacterial growth behavior of *Sal* and *Sal*@PST by monitoring the OD_600_ values, and expected, the growth of *Sal*@PST was much slower than that of the pristine *Sal* (Figure 2E).

While PST coating could decrease the potential side‐effects of bacteria, we expected such modification did not have any negative effect on anti‐tumor activities. Fortunately, tumor tissue is pathologically featured with slight acidic microenvironment, and we have demonstrated the pH‐responsive degradation of PST. We therefore reasoned that the PST coating could exfoliate from *Sal* surface at acidic tumor microenvironment, and thus recover the anti‐tumor activities. To verify this, we first studied the bacterial growth behavior of *Sal* and *Sal*@PST at different pHs (Figure 2E–G). Note that the activity of *Sal* was not damaged in these conditions (Figure S3). Although the growth of *Sal*@PST was inhibited at pH 7 due to *Sal* coating, an obvious recovery was observed at pH 6 and 5. For comparison, we also prepared the *Sal* coated with PDA (termed *Sal*@PDA) as control. *Sal* growth was also affected by PDA coating, which however was not restored upon pH decrease. This result highlighted the superiority of the PST over PDA as coating shell by virtue of its pH‐responsive degradation. As a result, the cytotoxicity of *Sal*@PST toward B16F10 tumor cells significantly enhanced after acidic pre‐treatment as evidenced by Annexin V‐FITC and PI double staining (Figure 2H and Figure S4). Therefore, degradation of PST coating could recover anti‐tumor effect of the bacteria.

In addition to directly damaging tumor cells, *Sal* could also promote anti‐tumor immunity via various mechanisms, such as DCs activation by the formation of gap‐junction between tumor cells and DCs to promote antigen presentation.^[^ ^43^, ^44^ ^]^ To study this mechanism, the expression of Cx43, the constituent protein of gap‐junction, was measured, and the cross‐talk between tumor cells and DCs via gap‐junction was monitored by using calcein‐AM (Figure 2I). As expected, the expression of Cx43 in both tumor cells and DCs were upregulated by *Sal* treatment, but this was not observed for *Sal*@PST (Figure 2J). Upon acidic pre‐treatment of *Sal*@PST, however, the upregulation effect on Cx43 can be restored. Because of Cx43 expression, the connection between tumor cells and DCs was promoted via the formation of gap‐junction, resulting in enhanced calcein‐AM transfer as evidenced by stronger fluorescence signal in DCs (Figure 2K and Figure S5) and higher degree of fluorescence colocalization (Figure 2L). The formation of gap‐junction is an important pathway to promote antigen presentation from tumor cells to DCs, resulting in DCs maturation. To further confirm this, the biomarkers of DCs maturation, including MHC II and CD86, were measured (Figure 2M,N). In line with the above observation, the number of MHC II and CD86 double positive cells significantly increased for *Sal* and *Sal*@PST plus acidic pre‐treatment. The greatly higher percentage of matured DCs for the *Sal*@PST pH 6.0 group compared to that of the *Sal* group may be derived from the proliferation of liberated *Sal* during acidic pre‐treatment. Collectively, all these demonstrated that PST coating could effectively minimize the potential side‐effects of *Sal*, but exfoliate from *Sal* surface after being delivered into acidic tumor microenvironment to reinstate the *Sal* for anti‐tumor therapy.

### 2.3 MnO_2_ as carrier to deliver Dz for enhanced stability, cell transportation, and self‐activated PD‐L1 mRNA cleavage

As described above, *Sal*@PST could combat tumor via different mechanisms, such as directly damaging tumor cells and promoting tumor antigens presentation. The matured DCs then activate and recruit cytotoxic T cells into tumor, which are the final effector cells for anti‐tumor immunity. However, the cunning tumor cells are adaptable to develop various immune escape mechanisms to bypass the T cells surveillance, and the most well‐known one is PD‐1/PD‐L1 immune checkpoint.^[^ ^45^ ^]^ To this end, various strategies have been developed to block or regulate PD‐L1, and several of them have realized clinical translation.^[^ ^46^, ^47^, ^48^ ^]^ In this work, we aimed to use Dz as a gene silencing tool to knock down PD‐L1 expression in tumor cells, which is expected to combine with *Sal*@PST for better immunotherapy. Specifically, an 8–17 mutant Dz was employed, which could effectively cleave target mRNA in biological matrix, and its catalytic activity can be significantly enhanced by transition metal cofactors such as Mn^2+^.^[^ ^34^ ^]^ However, just like other types of nucleic acid‐based tools, Dz as hydrophilic and negatively charged DNA polymer cannot freely penetrate cell membrane, which requires specific transfection reagents. We therefore developed manganese dioxide (MnO_2_) as carrier for Dz delivery.

We previously reported a facile method to prepare MnO_2_ nanoparticles by Mn^2+^ oxidation under alkaline condition using hyaluronic acid (HA) as template.^[^ ^49^ ^]^ To follow this idea, maleimide (Mal)‐modified HA was used as template to prepare MnO_2_, and then thiolated Dz was conjugated on nanoparticles via thiol‐Mal bond. HA‐Mal was synthesized through one‐step amide reaction (Figure S6A), which was characterized by both UV–vis and ^1^H NMR spectra (Figure S6B, S6C). The HA‐Mal‐templated MnO_2_ nanoparticles (termed HMN) were then prepared, which showed quite similar size and ζ potential to HA‐templated MnO_2_ (Figure S7). Then, various concentrations of thiolated Dz (with FAM fluorophore labeling) were incubated with the nanoparticles, and the conjugation rate was determined by measuring the fluorescence intensity of the supernatant (Figure S8), based on which the optimal feeding concentration of Dz turned out to be 20 μM.

**FIGURE 3:**
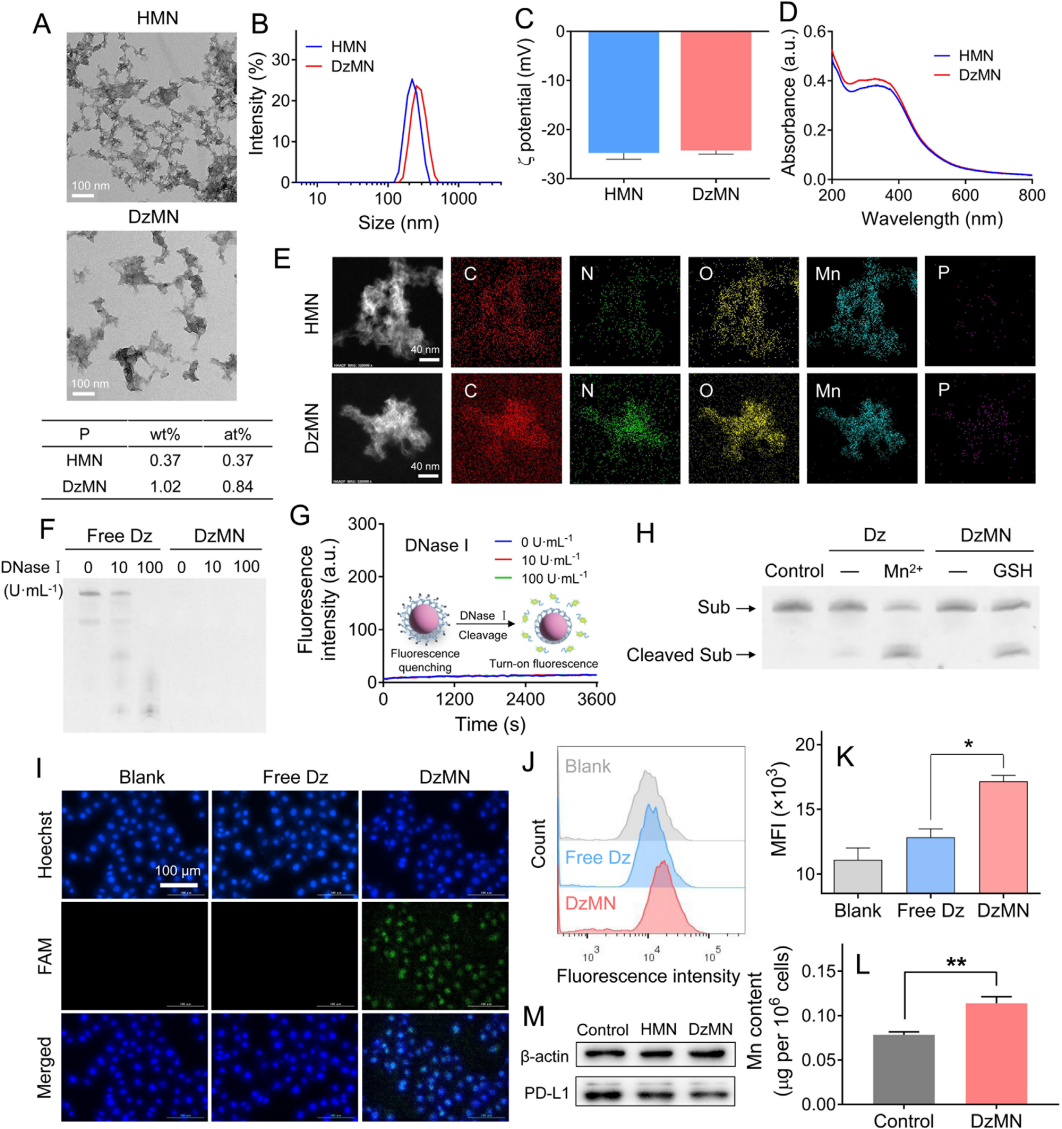
Preparation and characterizations of DNAzyme‐functionalized MnO_2_ nanoparticles (DzMN). (A) The transmission electron microscopy images. (B) The size distribution. (C) ζ potential (*n* = 3). (D) The UV–vis absorption spectrum and (E) element mapping of HMN and DzMN. (F) The PAGE images indicating the degradation of Dz in the free form or in DzMN after incubating with different concentrations of DNase I. (G) The dynamic fluorescence intensity of DzMN incubated with different concentrations of DNase I (*n* = 3). (H) The PAGE images of Dz cleavage activity under different conditions. (I) The fluorescence images and (J) the flow cytometry results of the cellular uptake of free Dz or DzMN. Scale bar = 100 μm. (K) The quantified results from (J) (*n* = 3). (L) The Mn content in B16F10 cells prior and after DzMN treatment were determined by ICP‐OES (*n* = 3). (M) The protein expression of PD‐L1 in B16F10 cells after different treatments detected by WB. Data were presented as mean ± SD. Statistical comparisons were performed using one‐way ANOVA for (K) and Student's *t*‐test for (L). *p < 0.05, **p < 0.01.

From the TEM images, HMN showed loose structure with certain adherence to each other, while the resulting Dz‐functionalized MnO_2_ nanoparticles (termed DzMN) had more rigid and compact structure with larger size (Figure 3A). Consistently, the DLS measurement also showed size increase post Dz conjugation (Figure 3B). Both HMN and DzMN exhibited a negative ζ potential of −25 mV (Figure 3C) but showed slightly higher UV–vis absorbance at 200–400 nm after Dz conjugation (Figure 3D), which can be attributed to the characteristic absorption of Dz as a type of nucleic acid. The ζ potential of DzMN with negatively charged Dz conjugation showed negligible difference compared to that of HMN, which can be attributed to the inherent strong negative ζ potential of HMN nanoparticles (approximately −25 mV). The structure component of the nanoparticles was further explored by element mapping (Figure 3E). HMN showed N and Mn signals ascribed to HA‐Mal and MnO_2_, respectively. For DzMN, P signal was also seen, further demonstrating the Dz conjugation.

Regarding biological applications of DNA, one critical concern is the enzymatic degradation during in vivo circulation. Fortunately, we previously showed that DNA conjugating on nanoparticles surface held much higher stability than its free counterpart.^[^ ^50^ ^]^ To demonstrate the protection effect, free Dz or DzMN was challenged with various concentrations of DNase I, and the degradation was analyzed by PAGE gel electrophoresis (note that the Dz was labeled with FAM fluorophore for visualization on gel band) (Figure 3F and Figure S9). For free Dz, obvious degradation bands were observed, and Dz was completely digested at 100 U·mL^−1^ DNase I. For DzMN, by contrast, no degradation band was seen even in presence of 100 U·mL^−1^ DNase I. Note that Dz was conjugated on nanoparticles surface, and thus it was retarded at sample loading well if it was not cleaved. Since MnO_2_ is a robust fluorescence quencher, the cleavage of Dz could also be probed by the turn‐on fluorescence. With DNase I treatment, however, no fluorescence recovery was observed over a period of 1 h (Figure 3G), demonstrating the resistance of Dz against enzymatic degradation after conjugation on MnO_2_. Besides protection effect, MnO_2_ could also serve as Mn^2+^ reservoir for Dz activation. To verify this, the PAGE gel assay was employed again to study the substrate cleavage. The Dz activity was dependent on Mn^2+^ concentration (Figure S10), and notably, the DzMN with GSH pre‐treatment also produced substantial substrate cleavage (Figure 3H and Figure S11), which can be attributable to GSH‐mediated degradation of MnO_2_ to generate Mn^2+^ for self‐activation of Dz (Figure S12).^[^ ^51^ ^]^

Next, the intracellular performance of DzMN was examined by using B16F10 cells. The nanoparticles were biocompatible and non‐toxic with equivalent Dz concentration up to 0.8 μM (Figure S13). Compared to free Dz, DzMN could effectively internalize into cells to produce bright fluorescence inside cells (Figure 3I–K). Along with this, we also quantified the internalization of the nanoparticles by measuring the Mn content (Figure 3L), which was significantly increased after DzMN treatment. The intracellularly delivered Mn^2+^ then activated Dz for PD‐L1 mRNA cleavage, resulting in the target protein downregulation (Figure 3M). Therefore, MnO_2_ was a versatile carrier, which could load Dz to improve its biological stability, promote its intracellular delivery, and importantly, act as Mn^2+^ reservoir to enable self‐activation of Dz for target mRNA cleavage.

### 2.4 The combination of *Sal*@PST and DzMN via interfacial DNA adsorption

**FIGURE 4:**
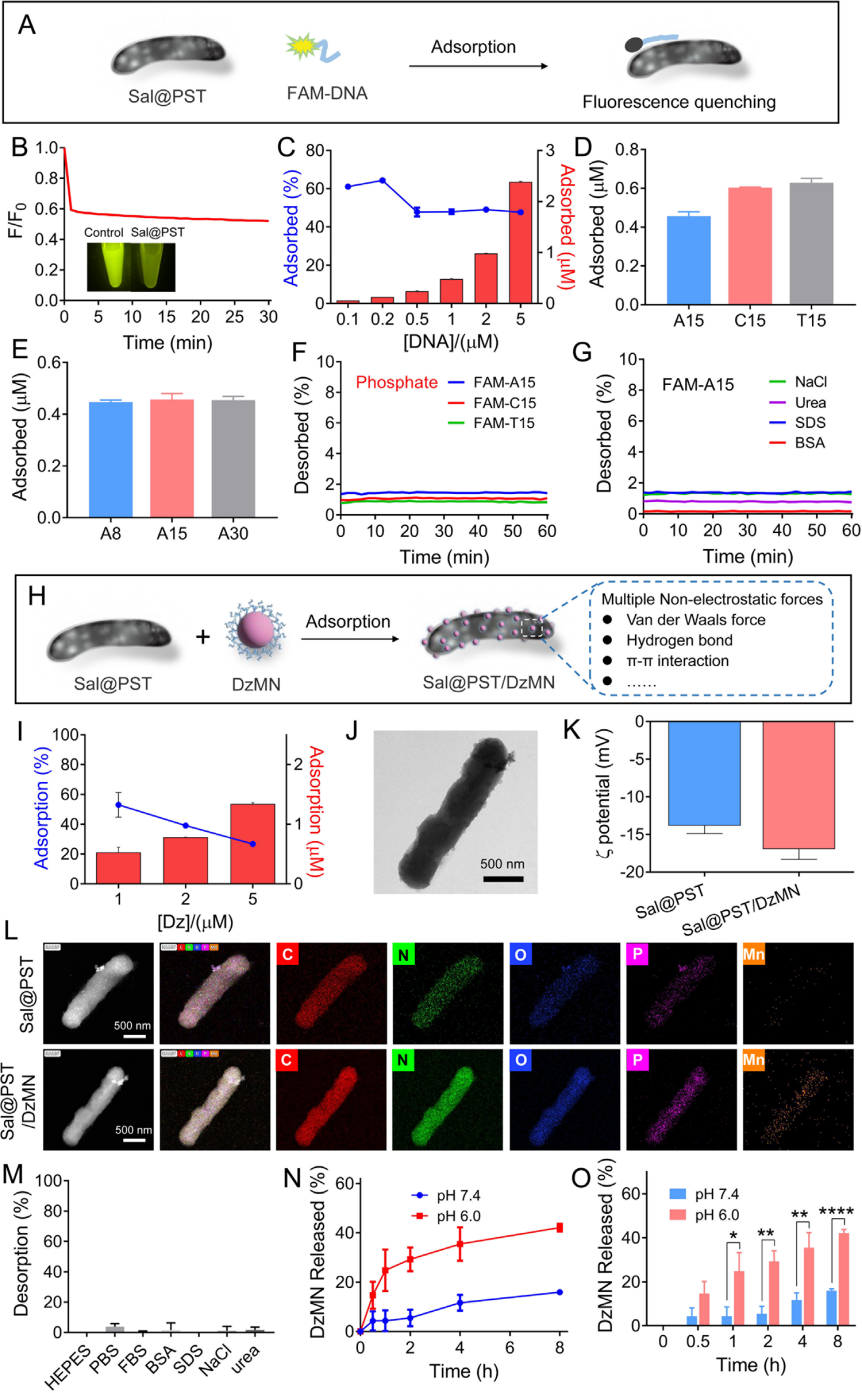
Preparation and characterizations of *Sal*@PST/DNAzyme‐functionalized MnO_2_ nanoparticles (DzMN). (A) Schematic illustration of the DNA adsorption by *Sal*@PST. (B) The adsorption kinetics of FAM‐labeled A15 by *Sal*@PST and the photographs of the supernatant post adsorption (*n* = 3). Effect of (C) DNA concentration, (D) DNA base, and (E) DNA length on adsorption rate by *Sal*@PST (*n* = 3). The DNA desorption kinetics from *Sal*@PST in the presence of (F) phosphate and (G) NaCl, urea, SDS, and BSA (*n* = 3). (H) Schematic illustration of the preparation of *Sal*@PST/DzMN. (I) The adsorption rate of DzMN on *Sal*@PST at different feeding concentrations (*n* = 3). (J) TEM image of *Sal*@PST/DzMN. Scale bar = 500 nm. (K) ζ potential of *Sal*@PST and *Sal*@PST/DzMN (*n* = 3). (L) Element mapping of *Sal*@PST and *Sal*@PST/DzMN. Scale bar = 500 nm. (M) The DzMN desorption in various media after incubation for 2 h (*n* = 3). (N, O) The DzMN release from *Sal*@PST/DzMN in different buffers (pH 7.4/6.0) (*n* = 3). Data were presented as mean ± SD. Statistical comparisons were performed using Student's *t*‐test for (O). *p < 0.05, **p < 0.01, ****p < 0.0001.

Given the complementary mechanism between DzMN and *Sal*@PST for anti‐tumor immunotherapy, we then integrated DzMN with *Sal*@PST to construct a combinatorial system. Previously, Liu and co‐workers showed that nanomaterials with densely functionalized DNA could stably adsorb on the surface of various materials to form hybrid structure,^[^ ^52^ ^]^ and the adsorption depended on the interfacial DNA interaction. DzMN was a type of DNA‐functionalized nanomaterial, which allowed its adsorption on *Sal*@PST. Like PDA, PST coating is good quencher,^[^ ^33^ ^]^ and thus its capability to adsorb DNA can be dynamically monitored by fluorescence decrease (Figure 4A). To simplify the system, an FAM‐labeled 15‐nt homo‐DNA with adenine base (A15) was used, and the fluorescence rapidly decreased upon mixing with *Sal*@PST in 2 min (Figure 4B), indicating effective DNA adsorption. The fluorescence was further confirmed by fluorescent images. We then changed the DNA concentration, and more DNA was loaded with higher feeding DNA up to 5 μM (Figure 4C), indicating high loading capacity of the PST surface. We then studied the effect of DNA bases (Figure 4D), in which poly‐C and poly‐T DNA showed considerably higher DNA loading, likely due to the critical contribution of nucleobases on adsorption.^[^ ^53^, ^54^, ^55^ ^]^ The DNA length, on the other hand, had no effect on DNA adsorption (Figure 4E). Finally, stability of the adsorbed DNA was tested by challenging with various competing ligands, including inorganic phosphate, NaCl, urea, SDS, and BSA (Figure 4F,G). Interestingly, none of these ligands could dissociate DNA from *Sal*@PST surface, indicating stable attachment for biological applications.

Having demonstrated the DNA adsorption of PST surface, we next prepared *Sal*@PST/DzMN via multi‐valent DNA adsorption (Figure 4H), and the resulting *Sal*@PST/DzMN was separated and collected by centrifugation (Figure S14). The loading capacity of DzMN was measured by UV–vis adsorption, in which the optimal feeding DzMN concentration turned out to be 5 μM (equivalent Dz concentration), resulting in adsorption ratio of ∼27% (Figure 4I). The color of *Sal*@PST changed from brownish red to pale brown post DzMN adsorption (Figure S15), and the *Sal*@PST surface with the satellite‐like structure was clearly seen from TEM (Figure 4J). After DzMN adsorption, the surface became more negatively charged because of dense DNA functionalization (Figure 4K), and the surface P and Mn were also observed based on element mapping (Figure 4L). All these results demonstrated the successful construction of *Sal*@PST/DzMN. We also studied the adsorption stability by adding different competing ligands (Figure 4M), and consistently, the DzMN could stably adsorb on *Sal*@PST to resist various displacement. Finally, the release of DzMN from *Sal*@PST was studied. Notably, a typical pH‐responsive release profile was observed (Figure 4N,O), which can be ascribed to the degradation of PST at acidic conditions. Therefore, *Sal*@PST/DzMN was stable during circulation while disintegrated under acidic tumor microenvironment to exert their respective functions for tumor therapy.

### 2.5 Anti‐tumor effect of *Sal*@PST/DzMN in vivo

**FIGURE 5:**
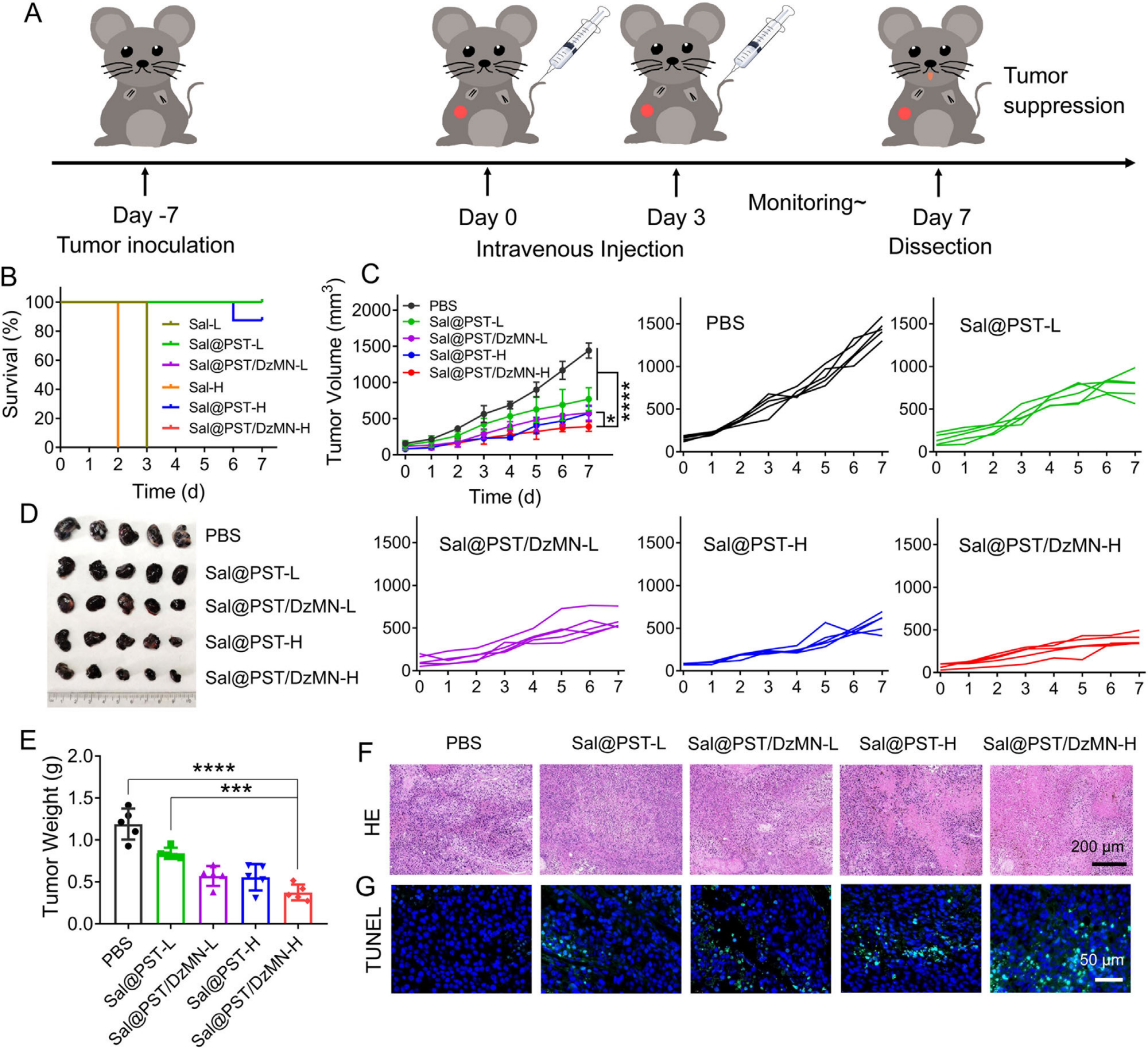
In vivo therapeutic efficacy. (A) Schematic illustrating the therapeutic schedule in B16F10 tumor‐bearing mice model. (B) The survival curves of mice after different treatments (*n* = 8). (C) The tumor growth curves of mice after different treatments (*n* = 5). (D) The photograph of tumors from mice after different treatments (*n* = 5). (E) The tumor weight of mice after different treatments (*n* = 5). (F) The H&E staining of tumor sections from mice after different treatments. Scale bar = 200 μm. (G) The TUNEL staining of tumor sections from mice after different treatments. Scale bar = 50 μm. Data were presented as mean ± SD. Statistical comparisons were performed using one‐way ANOVA for (C) and (E). *p < 0.05, ***p < 0.001, ****p < 0.0001.

Next, the in vivo behavior of *Sal*@PST/DzMN was evaluated by using B16F10 tumor‐bearing mice model. The biodistribution was first investigated after a single intravenous injection of DiR‐labeled *Sal*, *Sal*@PST, or *Sal*@PST/DzMN. Although *Sal* mainly distributed in the liver and spleen initially, as evidenced by fluorescence imaging and DiR quantification results (Figures S16 and S17), the amount of *Sal* in the tumor exceeded that in major organs over time due to the gradual clearance of *Sal* from normal organs and the continuous proliferation of *Sal* in the tumor microenvironment (Figure S18). Notably, the DiR signal in the *Sal*@PST and *Sal*@PST/DzMN groups was significantly stronger than that in the *Sal* group, indicating the reduced immune clearance of *Sal* facilitated by the surface coating. We then studied the anti‐tumor effect of *Sal*@PST/DzMN. The mice were randomly grouped and treated with PBS, *Sal*, *Sal*@PST, or *Sal*@PST/DzMN, and the therapeutic regimen was presented in Figure 5A. For all *Sal* groups, two doses termed L (for low dose) and H (for high dose) were treated. After injection of free *Sal*, all mice died in 3 days at both doses (Figure 5B), indicating severe toxicity of the bacteria owing to acute infection. For *Sal*@PST groups, by contrast, the survival rate significantly increased with only one mouse with high dose treatment dead at day 6, suggesting the detoxification of PST coating. And thus, only PST coating *Sal* groups were evaluated in the following studies. The anti‐tumor efficacy of each group was dynamically monitored by measuring tumor growth every day (Figure 5C), in which both *Sal*@PST and *Sal*@PST/DzMN could effectively suppress the tumor growth, and higher dose resulted in better outcome. Notably, *Sal*@PST/DzMN displayed better efficacy than *Sal*@PST at each dose, indicating the combinatorial effect between the bacteria and DzMN. We also extracted the tumor tissue after various treatments for direct observation and weighting (Figure 5D,E), and the same trend was obtained. To confirm the anti‐tumor effect, tumor tissues were further assayed by histological analysis. The H&E staining showed significant nuclear atrophy and cell necrosis, especially for *Sal*@PST/DzMN at high dose (Figure 5F), which also exhibited the strongest tumor apoptosis in TUNEL staining (Figure 5G). All these analyses demonstrated superior anti‐tumor activity of *Sal*@PST/DzMN.

### 2.6 Anti‐tumor mechanism of *Sal*@PST/DzMN by remodeling tumor microenvironment

**FIGURE 6:**
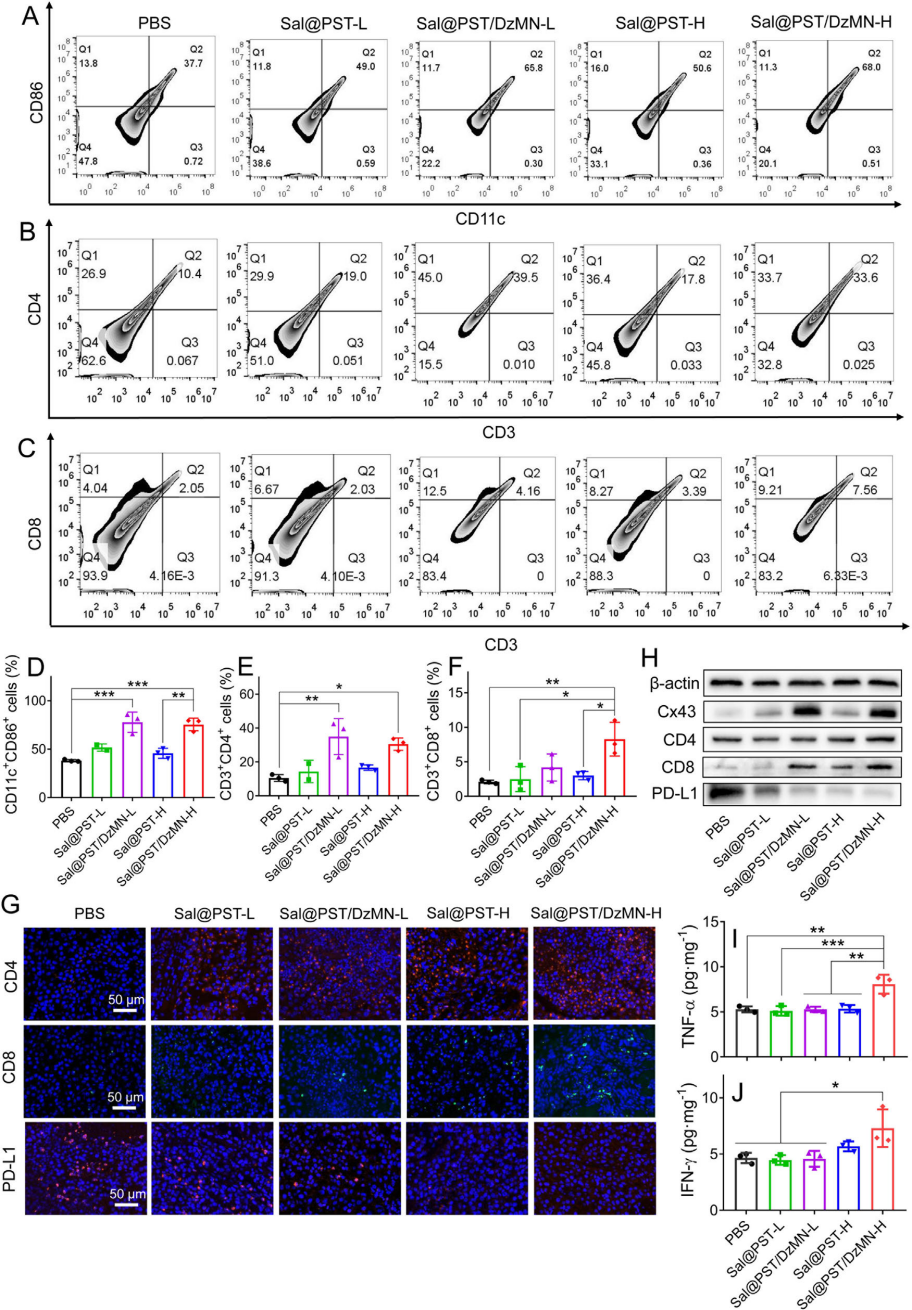
Assessment of tumor microenvironment remodeling. Representative flow cytometry results of (A) activated DCs (CD11c^+^CD86^+^), (B) CD4^+^ T cells (CD3^+^CD4^+^), and (C) CD8^+^ T cells (CD3^+^CD8^+^). (D–F) The quantified results of (A–C) (*n* = 3). (G) The immunofluorescence staining of CD4, CD8, and PD‐L1 of the tumor sections from mice after different treatments. Scale bar = 50 μm. (H) The protein expression of Cx43, CD4, CD8, and PD‐L1 in tumor tissues of mice after different treatments were detected by WB. The level of (I) TNF‐α and (J) IFN‐γ in tumor tissues of mice after different treatments (*n* = 3). Data were presented as mean ± SD. Statistical comparisons were performed using one‐way ANOVA for (D–F) and (I, J). *p < 0.05, **p < 0.01, ***p < 0.001.

Encouraged by the satisfactory anti‐tumor efficacy, we then explored the anti‐tumor mechanism. At cellular level, we have demonstrated the promotion of antigen presentation by *Sal* (Figure 2J–N) and PD‐L1 silencing effect of DzMN (Figure 3M), which lays the basis for their combinational anti‐tumor immunotherapy. To demonstrate this in vivo, the immunological change of tumor microenvironment was investigated after treatments. The immune cells were analyzed by flow cytometry, in which the activated DCs (identified by CD11c^+^CD86^+^) significantly increased (Figure 6A,D). This can be attributable to the formation of gap‐junction between tumor cells and DCs upon *Sal* treatment to promote antigen presentation, and this was confirmed by the increased expression of Cx43 (Figure 6H). Interestingly, the combination DzMN with *Sal*@PST could further promote DCs activation, which is benefited from the PD‐L1 suppression effect (Figure 6G,H and Figure S19).^[^ ^56^ ^]^ The activated DCs then primed T cell responses via presenting antigen and providing costimulation signals,^[^ ^57^ ^]^ thus resulting in the increase in the percentage of CD4^+^ and CD8^+^ T cells (Figure 6B,C,E,F). The effector T cells activation was further confirmed by both WB and immunofluorescence analyses of CD4 and CD8 proteins (Figure 6G,H). Notably, *Sal*@PST/DzMN groups showed significantly higher level of T cells infiltration and activation than *Sal*@PST, demonstrating the combinational effect of *Sal*@PST and DzMN for anti‐tumor immunity. In addition, the typical anti‐tumor cytokines, including tumor necrosis factor‐α (TNF‐α) and interferon‐γ (IFN‐γ), were measured in tumor (Figure 6I,J).^[^ ^58^ ^]^ As expected, significant increase of both cytokines was observed, suggesting the robust immune activation.

### 2.7 Biosafety evaluation

**FIGURE 7:**
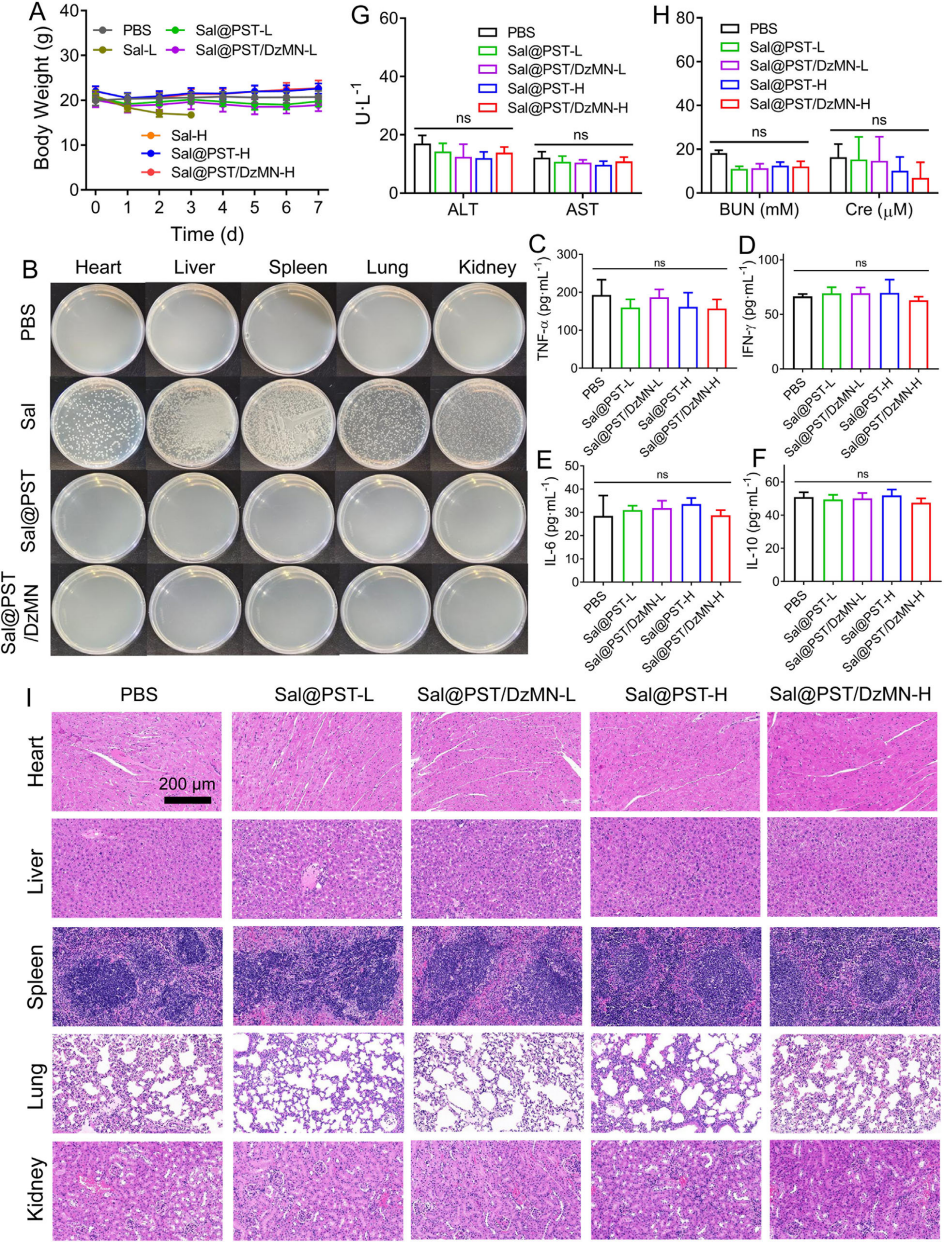
Evaluation of in vivo biosafety. (A) The body weight of mice during the treatments (*n* = 8). (B) Representative plate photographs of major organs homogenates after cultured at 37°C for 16 h. The serum level of (C) TNF‐α, (D) IFN‐γ, (E) IL‐6, and (F) IL‐10 of mice after different treatments (*n* = 5). (G, H) The serum level of biochemical indexes including ALT, AST, BUN, and Cre of mice post different treatments (*n* = 5). (I) The H&E staining of major organs from mice after different treatments. Scale bar = 200 μm. Data were presented as mean ± SD. Statistical comparisons were performed using one‐way ANOVA for (C–F) and (G, H). ns, not significant.

Biosafety is a crucial concern that needs to be considered for the in vivo application of bacteria. In the above study, free *Sal* showed high toxicity to directly kill mice, and the body weight dropped rapidly (Figure 7A). Upon PST coating, by contrast, the biocompatibility markedly increased, and no significant body weight change was observed. It has been shown that the toxicity of bacteria is mainly due to non‐specific spread from blood to normal organs to cause serious side‐effects. To explore this, we collected the major organs including heart, liver, spleen, lung, and kidneys of mice under sterile conditions at 24 h post *Sal* injection, which were homogenized and cultured on LB agar plate (Figure 7B). Notably, free *Sal* showed many bacteria colonies in different organs, consistent with the strong toxicity. For comparison, the PST coating groups merely produced any bacteria colony, attributable to the non‐degradable PST coating in normal organs to restrict bacteria growth. In addition, the several typical cytokines were also measured to evaluate the potential risk of bacteria‐induced cytokine storms (Figure 7C–F). All of the tested cytokines, including TNF‐α, IFN‐γ, interleukin‐6 (IL‐6), and IL‐10, did not show significant fluctuation. After treatment, the serum biochemical indexes, including ALT, AST, BUN, and Cre, were measured to evaluate the hepatic and renal function (Figure 7G,H). All these indexes were within normal range, suggesting no hepatotoxicity and nephrotoxicity. Moreover, the histological analysis of major organs was performed, and no obvious pathologic change was observed for all treatment groups (Figure 7I). Therefore, PST coating provides a highly promising modification to improve the therapeutic index of bacteria by virtue of its pH‐responsive exfoliation.

## 3 CONCLUSIONS

In summary, we reported a surface decoration strategy to solve the key limitations of *Sal* for tumor therapy. To improve the biocompatibility, *Sal* was coated with a pH‐responsive PST layer via a simple process. Such PST coating could detoxify bacteria both in vitro and in vivo via surface shield, which degrade in acidic tumor microenvironment to restore the anti‐tumor activities of *Sal*. To enhance efficacy, a self‐activated nanosystem was fabricated by conjugating Dz on MnO_2_ surface to form DzMN, which could effectively cleave PD‐L1 mRNA inside cells to block the immune checkpoint. DzMN was then attached on *Sal*@PST surface via interfacial DNA adsorption, forming a hybrid platform. This platform was able to target tumor tissue by hitchhiking the active tropism effect of *Sal*, and upon accumulation into tumor, PST degradation triggered the disintegration of the structure to release *Sal* and DzMN, which function combinatorially to regulate tumor immune microenvironment. As a result, an enhanced anti‐tumor immunotherapy was achieved with high biosafety. This work provides a promising strategy to improve the bacteria‐based tumor therapy via smart surface functionalization.

## AUTHOR CONTRIBUTIONS

**Lina Guo**: Conceptualization; investigation; writing—original draft. **Hao Chen**: Investigation. **Jinsong Ding**: Resources; supervision; funding acquisition. **Pengfei Rong**: Conceptualization; resources. **Ming Sun**: Writing—review and editing. **Wenhu Zhou**: Conceptualization; resources; supervision; writing—review and editing; funding acquisition.

## CONFLICT OF INTEREST STATEMENT

The authors declare no conflicts of interest.

## Supporting information

Additional supporting information can be found online in the Supporting Information section at the end of this article. Experimental details are provided in the Supporting Information.
Supporting information.

## Data Availability

The raw data and processed data required to reproduce these findings are available from the corresponding author upon request.
