# Supplementary material for: Surface engineering Salmonella with pH‐responsive polyserotonin and self‐activated DNAzyme for better microbial therapy of tumor
Source: Exploration (Beijing). 2023 Oct 5;3(6):20230017. doi: 10.1002/EXP.20230017 (PMC10742197; doi:10.1002/EXP.20230017)
Supplement: Supplementary file 1 — Additional supporting information can be found online in the Supporting Information section at the end of this article. Experimental details are provided in the Supporting Information. Supporting information. [file EXP2-3-20230017-s001.docx]

Supporting Information

**Surface Engineering Salmonella with pH-Responsive Polyserotonin and Self-Activated DNAzyme for Better Microbial Therapy of Tumor**

Lina Guo^1^, Hao Chen^2^, Jinsong Ding^1^, Pengfei Rong^3^, Ming Sun^4^ and Wenhu Zhou^1^*

^1^. Xiangya School of Pharmaceutical Sciences, Central South University, Changsha, Hunan, 410013, China

^2^. Department of Pathology, Shihezi University School of Medicine, Shihezi, 832003, China

^3^. Department of Radiology, The Third Xiangya Hospital, Central South University, Changsha, Hunan 410013, China

^4^. Division of Systems Pharmacology and Pharmacy, Leiden Academic Center for Drug Research, Leiden University, 2333 CC Leiden, The Netherlands

E-mail: [zhouwenhuyaoji@163.com](mailto:zhouwenhuyaoji@163.com)

**EXPERIMENTAL SECTION**

**Materials**

The serotonin (5-HT) hydrochloride was purchased from Yuanye Biotechnology Co., Ltd. (Shanghai, China). The sodium dodecyl sulfate (SDS), dimethyl sulfoxide (DMSO), Tween-80, ammonia water, sodium hydroxide (NaOH) and ethanol were bought from Sinopharm Chemical Reagent Co., Ltd. (Shanghai, China). The hyaluronic acid (HA) was obtained from Haixin Industrial Co., Ltd. (Qufu, China). The manganese chloride tetrahydrate (MnCl_2_·4H_2_O), bovine serum albumin (BSA) and glutathione (GSH) was bought from Aladdin (Shanghai, China). The N-(2-Aminoethyl) maleimide hydrochloride (Mal·HCl) was purchased from Rhawn Chemical Technology Co., Ltd. (Shanghai, China). The thiolated PD-L1 DNAzyme (Dz, 5’-SH-TCAGAGGGTTCAACATCCGAGCCGGTCGAAGCTTACGTCTCCTCG-3’), FAM-labelled counterpart and corresponding substrate (5’-CGAGGAGACGTAAGCTrArGTGTTGAACCCTCTGA-3’) were synthesized by Sangon Biotech Co., Ltd. (Shanghai, China). The deoxyribonuclease Ⅰ (DNase Ⅰ) was bought from Invitrogen Life Technologies (California, USA). The Roswell Park Memorial Institute (RPMI)-1640 medium, Dulbecco’s Modified Eagle’s Medium (DMEM), fetal bovine serum (FBS) and penicillin-streptomycin solution were purchased from Gibco Life Technologies, Inc. (Grand Island, NY, USA). The LB broth, Cell Apoptosis Detection Kit, Mouse Peripheral Blood Neutrophil Isolation Kit, methyl thiazolyl tetrazolium (MTT), Hoechst 33342 and 4′,6-diamidino-2-phenylindole (DAPI) were bought from Solarbio Biotech Co., Ltd. (Beijing, China). The LB agar powder was obtained from Sangon Biotech Co., Ltd. (Shanghai, China). The Calcein-AM was purchased from Beyotime Biotechnology (Shanghai, China). The BCA Protein Quantification Kit was obtained from Dingguo Changsheng Biotechnology Co., Ltd. (Beijing, China). The primary antibody against connexin 43 (Cx43) was obtained from Proteintech Group (Chicago, USA) and the antibody against programmed death ligand-1 (PD-L1), CD4 and CD8α were obtained from ABclonal Technology Co., Ltd. (Wuhan, China). The fluorescence-labelled antibody against CD11c, CD86, CD3, CD4 and CD8 were obtained from BioLegend (California, USA). The FITC-labelled antibody against MHCⅡ was provided by Bioss (Beijing, China). The DiR dye was purchased from Maokang Biotechnology Co., Ltd. (Shanghai, China). The ELISA kits for tumor necrosis factor-α (TNF-α), interferon-γ (IFN-γ), interleukin-6 (IL-6) and IL-10 were bought from Meimian Industrial Co., Ltd. (Jiangsu, China). The assay kits for alanine transaminase (ALT), aspartate transaminase (AST), blood urea nitrogen (BUN) and creatinine (Cre) were obtained from Jiancheng Bioengineering Institute (Nanjing, China).

**Bacteria and cell culture**

The *Salmonella* typhimurium strain ΔppGpp (denoted as *Sal*) was kindly provided by Jinhai Zheng from Hunan University. The bacteria were inoculated into LB broth and cultured at 37°C in a shaking incubator at a shaking speed of 180 rpm. Afterwards, the cultures were transferred for several times and finally collected by centrifugation at 3000 rpm for 5 min. The settled bacteria were resuspended in sterile PBS for further experiments.

The murine macrophage cells (RAW264.7), murine bone marrow-derived dendritic cells (DC2.4), murine melanoma cells (B16F10) and human embryonic kidney cells (HEK293T) were obtained from Xiangya cell center (Changsha, China). The RAW264.7 and DC2.4 cells were cultured in RPMI-1640 medium while B16F10 and HEK293T cells were cultured in DMEM medium. All the medium were supplemented with 10% FBS and 1% penicillin-streptomycin solution, and the cells were placed in an incubator at 37°C with 5% CO_2_.

**Preparation and characterizations of *Sal*@PST/DzMN**

***Preparation and characterizations of Sal@PST***

The bacteria was collected and resuspended to a concentration of 1.6×10^9^ CFU·mL^-1^ according to the optical density (OD) values at 600 nm. Afterwards, Tween-80 (10 mM), ethanol and 5-HT aqueous solution (2 mg·mL^-1^) were mixed with bacterial suspension (50 μL), followed by pH adjustment with ammonia water and then stirred at 37°C for 4 h. The resultant *Sal*@PST was collected by centrifugation at 3000 rpm for 5 min, washed for several times and resuspended. The size and morphology of *Sal*@PST were characterized by dynamic light scattering (DLS) with scattering angle of 90° and transmission electron microscopy (TEM). The UV-Vis and FT-IR spectra were also measured to demonstrate the successful surface decoration. The pH-responsive degradation behavior of PST was investigated by measuring the UV-Vis spectra of supernatant after incubating *Sal*@PST in buffers with various pH (7.4/6.0/5.0) overnight.

***Preparation and characterizations of DzMN***

First, HA-Mal was synthesized according to the previous reports.^[1]^ Briefly, HA (0.5 mmol, 200.5 mg), EDC (0.6 mmol, 115.0 mg) and NHS (0.6 mmol, 69.1 mg) were dissolved in MES buffer (0.1 M, pH 4.5, 22 mL) and stirred at room temperature for 30 min. The Mal·HCl (1 mmol, 176.6 mg) dissolved in water (1 mL) was then added dropwise and allowed to react for 24 h. After the reaction, the mixture was dialyzed (MWCO = 3500 Da) against water for 3 d and then lyophilized for 24 h. The UV-Vis and ^1^H NMR spectra (with deuterated DMSO as solvents) were measured to prove the successful synthesis of HA-Mal.

Second, HA-Mal-templated MnO_2_ nanoparticles (HMN) were prepared. Briefly, NaOH (1 M, 80 μL) and MnCl_2_ (20 mg·mL^-1^, 40 μL) were successively added into HA-Mal solution (5 mg·mL^-1^, 4 mL) under stirring, followed by sonication for 10 min. The HMN was collected by centrifugation at 20000 rpm for 10 min. The size and ζ potential were characterized by Malvern Zeta Sizer Nano series (Nano ZS, Malvern Instruments, UK).

Finally, DzMN was prepared by conjugating thiolated PD-L1 Dz onto HMN. The HMN was resuspended in HEPES (10 mM, pH 7.4), with thiolated PD-L1 Dz (20 μM) added and incubated at room temperature overnight. The resultant DzMN was collected by centrifugation at 20000 rpm for 10 min. To characterize the conjugation ratio, the FAM-labelled thiolated PD-L1 Dz was used and the fluorescence intensity of the supernatant after incubation was measured. The size and ζ potential of DzMN prepared at optimal Dz concentration were characterized by Malvern Zeta Sizer Nano series (Nano ZS, Malvern Instruments, UK). After depositing the samples onto the carbon film supported copper grids without any post-processing, the morphologic observation and element mapping were performed using TEM. The protective effect of DzMN against enzymatic degradation was investigated by incubating DzMN with different concentrations of DNase Ⅰ at 37°C for 1 h and then detecting by fluorescence measurement and polyacrylamide gel electrophoresis (PAGE). The cleavage activity of Dz was investigated by incubating free Dz or DzMN with the substrate at 37°C for 1 h and then detected by PAGE.

***Preparation and characterizations of Sal@PST/DzMN***

The *Sal*@PST was incubated with DzMN (Dz equivalent concentration of 5 μM) at 37°C for 2 h, followed by centrifugation at 3000 rpm for 5 min to obtain *Sal*@PST/DzMN. The element mapping was performed to demonstrate the successful adsorption. The absorbance of supernatant at 340 nm was further measured to determine the adsorption capacity. The adsorption stability was investigated by measuring the absorbance of supernatant at 340 nm after incubating *Sal*@PST/DzMN in various displacing media (HEPES, PBS, FBS, BSA, SDS, NaCl and urea) for 2 h.

**DNA adsorption mechanisms**

For a typical experiment, *Sal* or *Sal*@PST were incubated with FAM-labelled A15 (1 μM) in HEPES (10 mM, pH 7.4) and the fluorescence intensity was dynamically monitored every minute for 30 min by a microplate reader (Infinite M200 PRO, TECAN, Austria). The supernatant post incubation was photographed under ultraviolet light, with free A15 (1 μM) as control. The DNA adsorption capacity of *Sal*@PST was further investigated by feeding various concentrations of FAM-labelled A15 (0.1, 0.2, 0.5, 1, 2 and 5 μM). Then, the adsorption of DNA with different sequence (A15, C15 and T15) and length (A8, A15 and A30) was investigated. For DNA displacement studies, desorption reagents (10 μL) were added into the prepared *Sal*@PST/DNA (90 μL) and the fluorescence intensity was dynamically monitored every 2 minutes for 60 min by a microplate reader (Infinite M200 PRO, TECAN, Austria). The desorption reagents included phosphate (100 mM, pH 7.4), NaCl (5 M), urea (16 M), SDS (10 mg/mL) and BSA (50 mg/mL).

**pH-responsive DzMN release**

The release profile of DzMN from *Sal*@PST/DzMN at different pH was investigated by incubating *Sal*@PST/DzMN in different buffers (pH 7.4/6.0) at 37°C with a constant shaking of 100 rpm. The samples were taken at pre-determined timepoints and the supernatant was collected post centrifugation at 3000 rpm for 5 min, followed by centrifugation at 20000 rpm for 10 min to precipitate the released DzMN. The precipitate was then resuspended in water and the absorbance at 340 nm was measured. The cumulative *in vitro* release percentage was calculated accordingly.

**Bacterial viability**

The bacterial viability of *Sal* at different pHs was investigated by SYTO 9/PI double staining. The *Sal* was collected, inoculated into LB broth (pH 7.0/6.0/5.0 adjusted by HCl) and incubated overnight. These samples were collected by centrifugation and resuspended in saline. Then, SYTO 9 and PI (1.5 μL each) were added into the bacterial suspensions (1 mL) and incubated in the dark for 15 min, followed by washing with saline for three times. The fluorescent microscopy was used to image the samples.

**Bacterial growth curve**

The *Sal*, *Sal*@PST and *Sal*@PDA (*Sal* with polydopamine coating prepared by replacing 5-HT with dopamine) were collected, inoculated into LB broth (pH 7.0/6.0/5.0 adjusted by HCl) and placed in a microplate reader (Infinite M200 PRO, TECAN, Austria) with constant shaking and constant temperature of 37°C. The OD values at 600 nm were measured every two hours and the bacterial growth curves were drawn.

**Immune clearance *in vitro***

The bacterial phagocytosis by macrophages and neutrophils were investigated by using *Sal* expressing green fluorescence protein (GFP). Briefly, RAW264.7 cells were seeded in a 24-well plate and incubated overnight. *Sal* or *Sal*@PST was added for 1.5 h incubation, followed by washing with PBS for three times and resuspending in PBS. The fluorescence intensity was determined by flow cytometry. The neutrophils were isolated from the peripheral blood of mice and labelled with Cell Tracker Deep Red at 37°C for 30 min. Then, they were incubated with *Sal* or *Sal*@PST for 1.5 h and the samples were analyzed by flow cytometry.

**Cell apoptosis study**

The B16F10 or HEK293T cells were seeded in 6-well plates at a density of 2×10^5^ cells per well and incubated overnight. *Sal* or *Sal*@PST pre-incubated in buffer (pH 7.4/6.0) overnight were added for 24 h incubation. After that, the cells were collected, washed with PBS for three times and resuspended in binding buffer (1×, 100 μL). Then, Annexin-FITC (5 μL) and PI (5 μL) were successively added into the suspensions and the samples were analyzed by flow cytometry.

**Gap-junction formation**

***Cx43 expression***

The expression of Cx43 was detected by Western Blot (WB). Briefly, the B16F10 or DC2.4 cells were seeded in 6-well plates at a density of 3×10^5^ cells per well and incubated overnight. *Sal* or *Sal*@PST pre-incubated in buffer (pH 7.4/6.0) overnight were added for 4 h incubation. After that, the cells were washed with PBS for three times and fresh medium supplemented with gentamycin (50 μg·mL^-1^) was added for further incubation. After 20 h, the total protein was extracted with RIPA lysis buffer and quantified with BCA Protein Quantification Kit. The protein concentration of each sample was adjusted to the same and then denatured at 95°C for 10 min. The samples with equal volumes were loaded on sodium dodecyl sulfate-polyacrylamide gel electrophoresis (SDS-PAGE) to be separated and then transferred onto the PVDF membrane. After blocking with 5% non-fat milk, the membranes were incubated with primary antibody against Cx43 at 4°C overnight and then incubated with horseradish peroxidase (HRP)-conjugated secondary antibody for 1 h, followed by visualization by enhanced chemiluminescence (ECL).

***Fluorescent dye transfer***

For flow cytometry analysis, the B16F10 and DC2.4 cells were seeded in 6-well plates at a density of 3×10^5^ cells per well and incubated overnight. *Sal* or *Sal*@PST pre-incubated in buffer (pH 7.4/6.0) overnight were added for 4 h incubation. After that, the cells were washed with PBS for three times and fresh medium supplemented with gentamycin (50 μg·mL^-1^) was added for further incubation. After 20 h, the B16F10 and DC2.4 cells were collected and stained with Calcein-AM (2 μM) and DDAO (10 μM), respectively. Then, they were co-incubated in the cell culture medium (B16F10:DC2.4 = 2:1) for 1 h. The cells were then collected, resuspended in PBS and analyzed by flow cytometry. For fluorescence imaging, the cells were treated as described above. After the treatment, the B16F10 cells were stained with Calcein-AM (2 μM) and then co-incubated with the DC2.4 cells stained with DAPI (1 μg·mL^-1^). After 1 h, the images were taken by a fluorescent microscope.

***DCs maturation***

The B16F10 and DC2.4 cells were seeded in 12-well plates at a density of 1×10^5^ cells per well and incubated overnight. *Sal* or *Sal*@PST pre-incubated in buffer (pH 7.4/6.0) overnight were added for 4 h incubation. After that, the cells were washed with PBS for three times and fresh medium supplemented with gentamycin (50 μg·mL^-1^) was added for further incubation. After 20 h, the B16F10 and DC2.4 cells were collected and further co-incubated for 24 h. Then, the cells were collected and incubated with anti-MHCⅡ-FITC and anti-CD86-PE at 4°C for 0.5 h. The samples were then analyzed by flow cytometry.

**Cell viability study**

The B16F10 cells were seeded in 96-well plate at a density of 5×10^3^ cells per well and incubated overnight. Then, different concentrations of DzMN were added for 48 h incubation. After that, the medium was discarded and the cells were washed with PBS for three times. After further culturing with MTT (0.5 mg·mL^-1^) for 4 h, the medium was discarded and the generated formazan was dissolved by DMSO (100 μL). The absorbance at 490 nm was measured by a microplate reader (Infinite M200 PRO, TECAN, Austria).

**Cellular uptake study**

For fluorescence imaging, the B16F10 cells were seeded in 24-well plate and incubated overnight. Then, the cells were treated with FAM-labelled PD-L1 Dz or as-prepared FAM-DzMN (Dz equivalent concentration of 400 nM). After 4 h, the cells were washed with PBS for three times and fixed with 4% paraformaldehyde for 15 min. The cellular uptake was observed with a fluorescent microscope after staining cell nuclei with Hoechst 33342. For flow cytometry analysis, the cells were collected after the treatment, resuspended in PBS and analyzed by flow cytometry. For Mn content quantification, the B16F10 cells were treated with DzMN (Dz equivalent concentration of 400 nM) for 24 h. After that, the cells were collected, counted and then digested with concentrated nitric acid. The digested samples were diluted for 10 times with distilled water and the Mn content was determined by inductively coupled plasma optical emission spectrometer (ICP-OES).

**PD-L1 silencing effect**

The B16F10 cells were seeded in 6-well plates at a density of 3×10^5^ cells per well and incubated overnight. Then, the cells were treated with HMN or DzMN (Dz equivalent concentration of 400 nM) for 48 h. The total protein was extracted and WB was performed as described above by using primary antibody against PD-L1.

**Animal models**

The male C57BL/6 mice (6-8 weeks old) were purchased from Hunan SJA Laboratory Animal Co., Ltd. (Changsha, China). All the animal experiments were approved by Central South University with the assigned approval/accreditation number of 2021-XMSB-0198. To establish the melanoma tumor models, 1×10^6^ B16F10 cells suspended in PBS were subcutaneously inoculated into the axilla of mice. The tumor growth was monitored closely, with tumor volume calculated by the formula of V = (length×width^2^)/2.

***In vivo* biodistribution study**

When the tumor volume reached ~100 mm^3^, the mice were randomly divided into four groups and intravenously injected with PBS, DiR-labelled *Sal*, *Sal*@PST or *Sal*@PST/DzMN at a *Sal* dose of 2×10^7^ CFU per mouse. The *in vivo* fluorescence imaging was performed using IVIS system at 8 h and 24 h post injection. Then, the mice were sacrificed and their major organs and tumors were collected for *ex vivo* imaging. These tissues were then homogenized in methanol, with supernatant collected post centrifugation at 10000 rpm for 5 min. The fluorescence intensity was measured by a microplate reader (Infinite M200 PRO, TECAN, Austria) for DiR quantification (Ex: 748 nm, Em: 780 nm).

***In vivo* antitumor study**

When the tumor volume reached ~100 mm^3^, the mice were randomly divided into seven groups and intravenously injected with PBS, *Sal*, *Sal*@PST or *Sal*@PST/DzMN at a *Sal* dose of 6×10^6^ CFU or 2×10^7^ CFU per mouse at day 0 and day 3. The tumor volumes of mice were recorded every day. The mice were sacrificed at day 7, with major organs (heart, liver, spleen, lung and kidneys), tumors and the blood collected for further analysis. The photograph of tumors was taken and the tumor weight were recorded.

**Histological analysis**

The tumor tissues were fixed with paraformaldehyde, followed by paraffin embedding and slicing. The sections were imaged with a light microscope after being stained with hematoxylin & eosin (H&E). The TUNEL staining was also performed referring to the instructions and then imaged with a fluorescent microscope.

**Immunological effect**

The immune cells infiltration in tumors were investigated by flow cytometry, immunofluorescence and WB. For flow cytometry, the tumors were grinded to filter through the cell strainers of 40 μm to obtain single cell suspensions, followed by red blood cell lysis with Red Blood Cell Lysis Buffer. The cells were then incubated with the fluorescence-labelled antibodies at 4°C for 20 min after blocking with CD16/32 blocking reagent. For DCs maturation, the cells were incubated with anti-CD11c-APC and anti-CD86-PE. For T cells activation, the cells were incubated with anti-CD3-FITC, anti-CD4-APC and anti-CD8-PE. After the incubation, the samples were measured by flow cytometry. For immunofluorescence, the tumor sections were incubated with the primary antibody against PD-L1, CD4 and CD8 at 4°C overnight and then incubated with the fluorescence-labelled secondary antibodies for 1 h. The images were taken by a fluorescent microscope with cell nuclei stained with DAPI. For WB, the tumor tissues were homogenized in RIPA lysis buffer (100 mg:1 mL) and further incubated at 4°C for 1 h, with supernatant collected post centrifugation at 13000 rpm for 15 min to obtain the protein samples. The WB was performed as described above by using primary antibodies against Cx43, PD-L1, CD4 and CD8. To confirm the PD-L1 silencing effect, the mRNA levels of PD-L1 in tumor tissues were investigated by RT-PCR post extracting total RNA by TRIzol reagent.

The immune-related cytokines were determined by ELISA. Briefly, the tumor tissues were homogenized in ice-cold PBS and then centrifuged at 13000 rpm for 15 min. The levels of TNF-α and IFN-γ in the supernatant were measured by the corresponding ELISA kits.

**Safety evaluation**

The body weight of mice was monitored every day during the treatment. The major organs were fixed with paraformaldehyde, sliced and then stained with H&E. The sections were observed under a light microscope. The blood was centrifuged to obtain serum. The serum levels of biochemical indexes including ALT, AST, BUN and Cre were measured by corresponding assay kits. In addition, the serum levels of various cytokines including TNF-α, IFN-γ, IL-6 and IL-10 were determined by corresponding ELISA kits.

**Bacterial proliferation *in vivo***

When the tumor volume reached ~100 mm^3^, the mice were intravenously injected with *Sal*, *Sal*@PST or *Sal*@PST/DzMN at a *Sal* dose of 6×10^6^ CFU per mouse. The mice were sacrificed at 24 h post injection and their major organs (heart, liver, spleen, lung and kidneys) were collected, weighed and homogenized in sterile PBS (100 mg:0.9 mL). The homogenates were diluted by 100-fold and plated on solid LB agar plates. The colonies on the plates were observed after culturing at 37°C overnight.

**Statistical analysis**

All the quantitative data were represented as mean ± standard deviation (SD). The differences between two groups and among multiple groups were assessed by the Student’s t-test and one-way analysis of variance (ANOVA), respectively. The significance was defined at the following thresholds: **p* < 0.05, ***p* < 0.01, ****p* < 0.001, *****p* < 0.0001.

**Supplementary figures:**

**
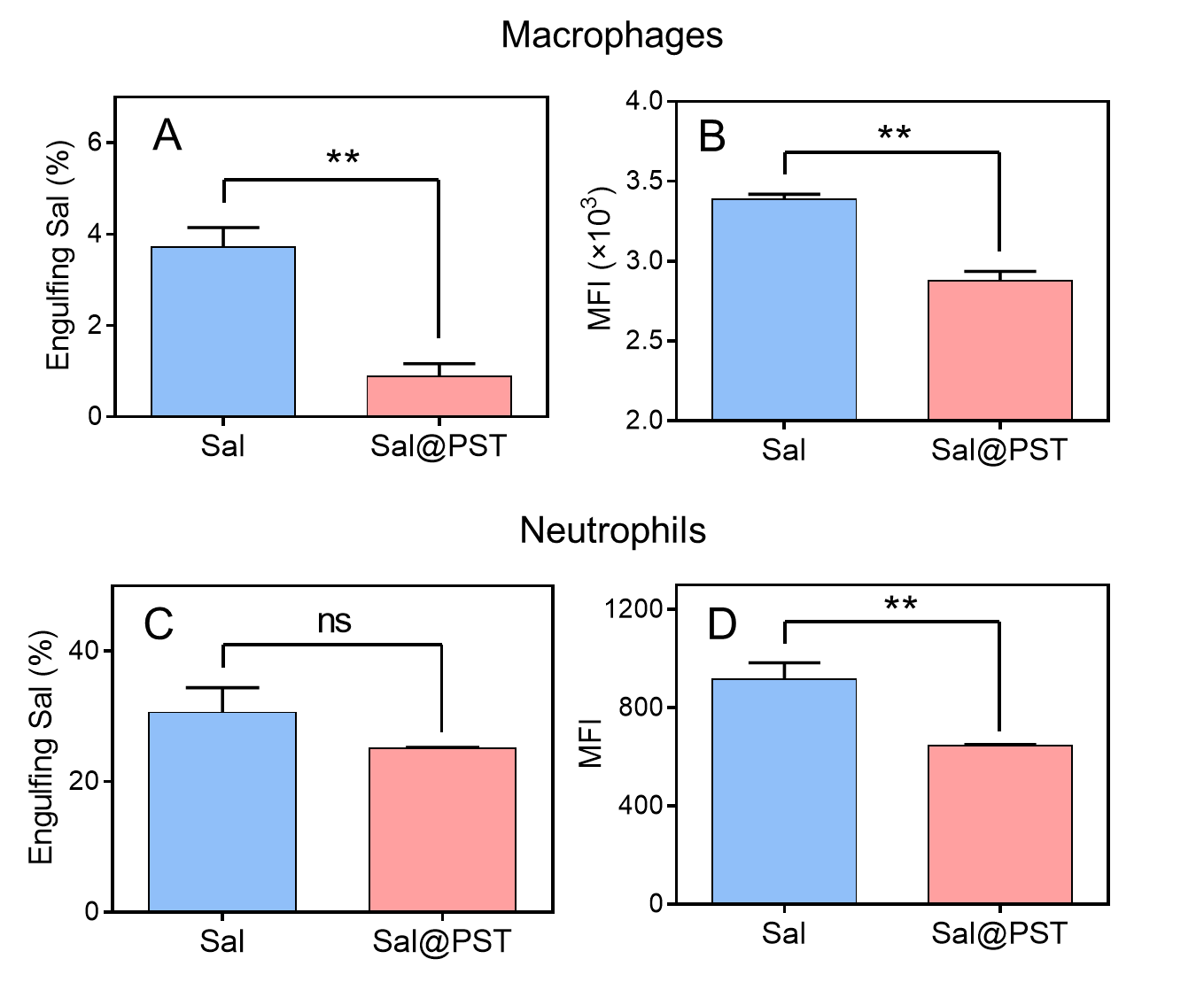
**

**Figure S1.** (A) The percentage of macrophages engulfing *Sal* (n=3). (B) The quantification of the mean fluorescence intensity (MFI) of the phagocytized *Sal* in macrophages (n=3). (C) The percentage of neutrophils engulfing *Sal* (n=3). (D) The quantification of the MFI of the phagocytized *Sal* in neutrophils (n=3). Data were presented as mean ± SD. Statistical comparisons were performed using Student’s t-test for (A-D). ns, not significant, ***p* < 0.01.


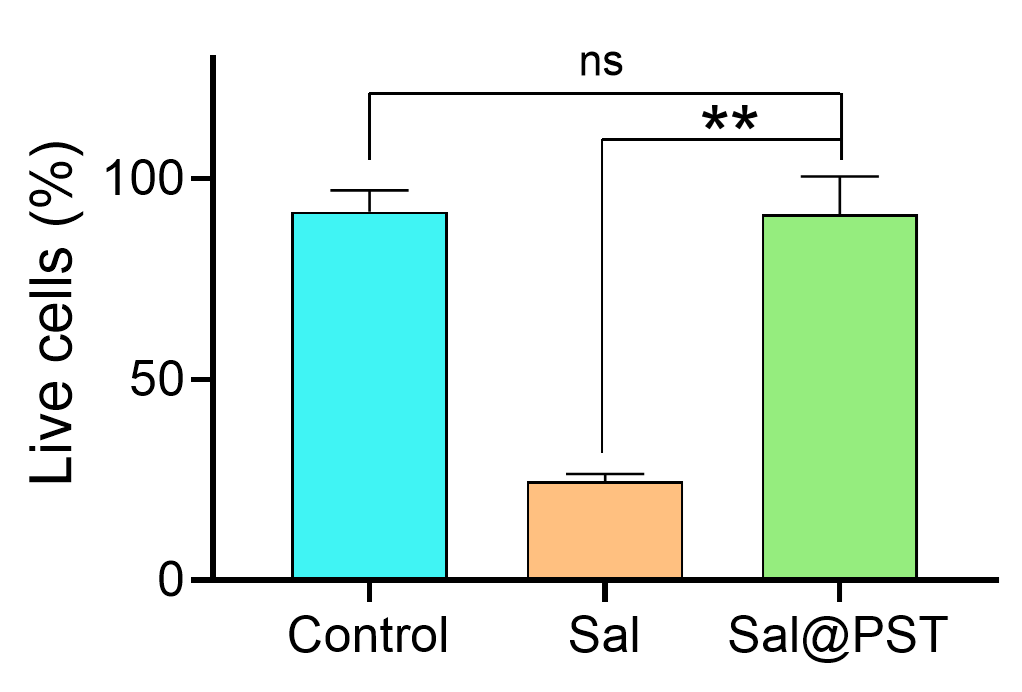


**Figure S2.** The percentage of live HEK293T cells after different treatments (n=3). Data were presented as mean ± SD. Statistical comparisons were performed using one-way ANOVA. ns, not significant, ***p* < 0.01.


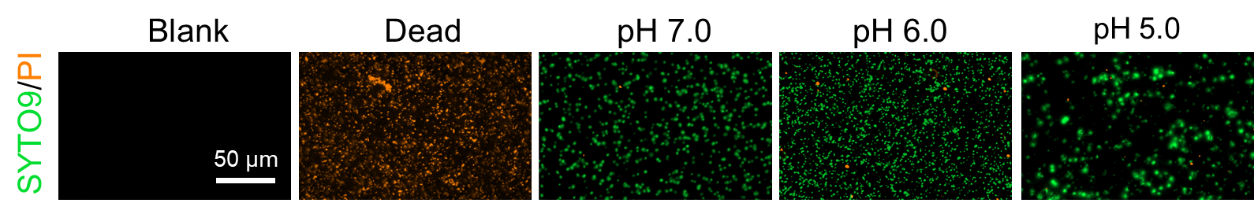


**Figure S3.** The live/dead bacterial staining by SYTO9/PI double staining, for which live bacteria would be stained green while dead bacteria would be stained red. The majority of bacteria exhibited green fluorescence at all tested pH values, suggesting that *Sal* could tolerate such acidic conditions. Scale bar = 50 μm.


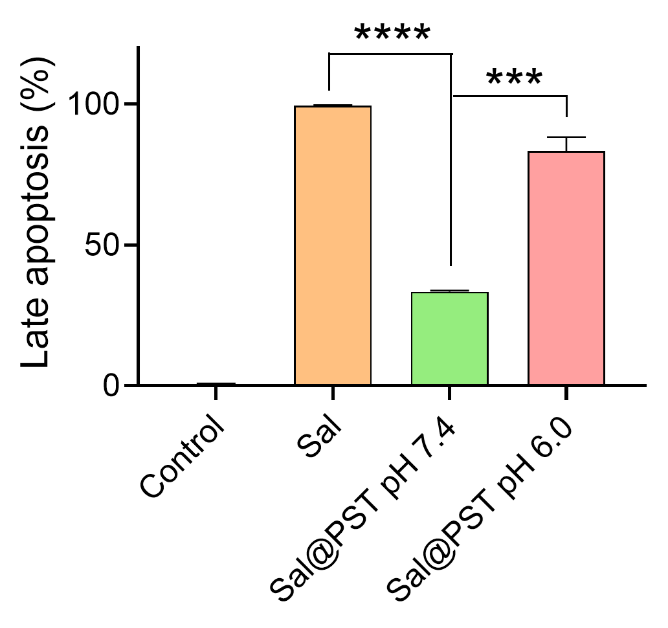


**Figure S4.** The percentage of late apoptotic cells for B16F10 cells post different treatments (n=3). Data were presented as mean ± SD. Statistical comparisons were performed using one-way ANOVA. ****p* < 0.001, *****p* < 0.0001.


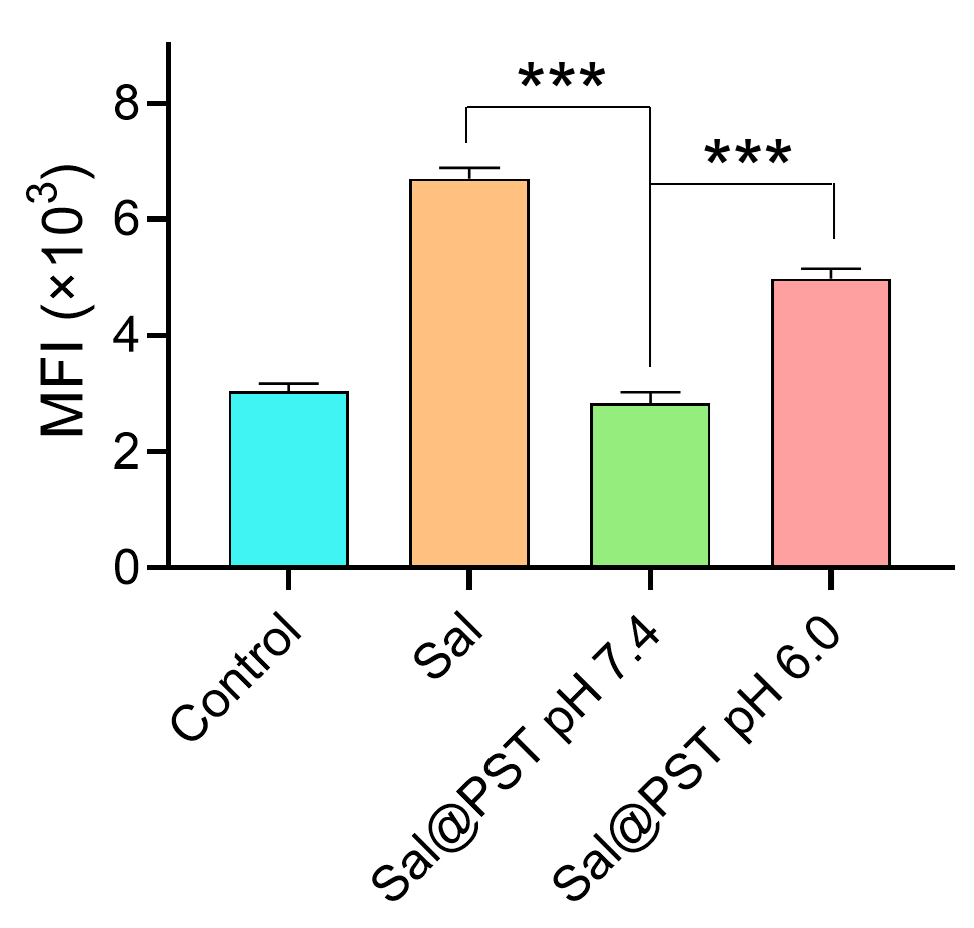


**Figure S5.** The MFI of Calcein-AM in DC2.4 cells post different treatments (n=3). Data were presented as mean ± SD. Statistical comparisons were performed using one-way ANOVA. ****p* < 0.001.


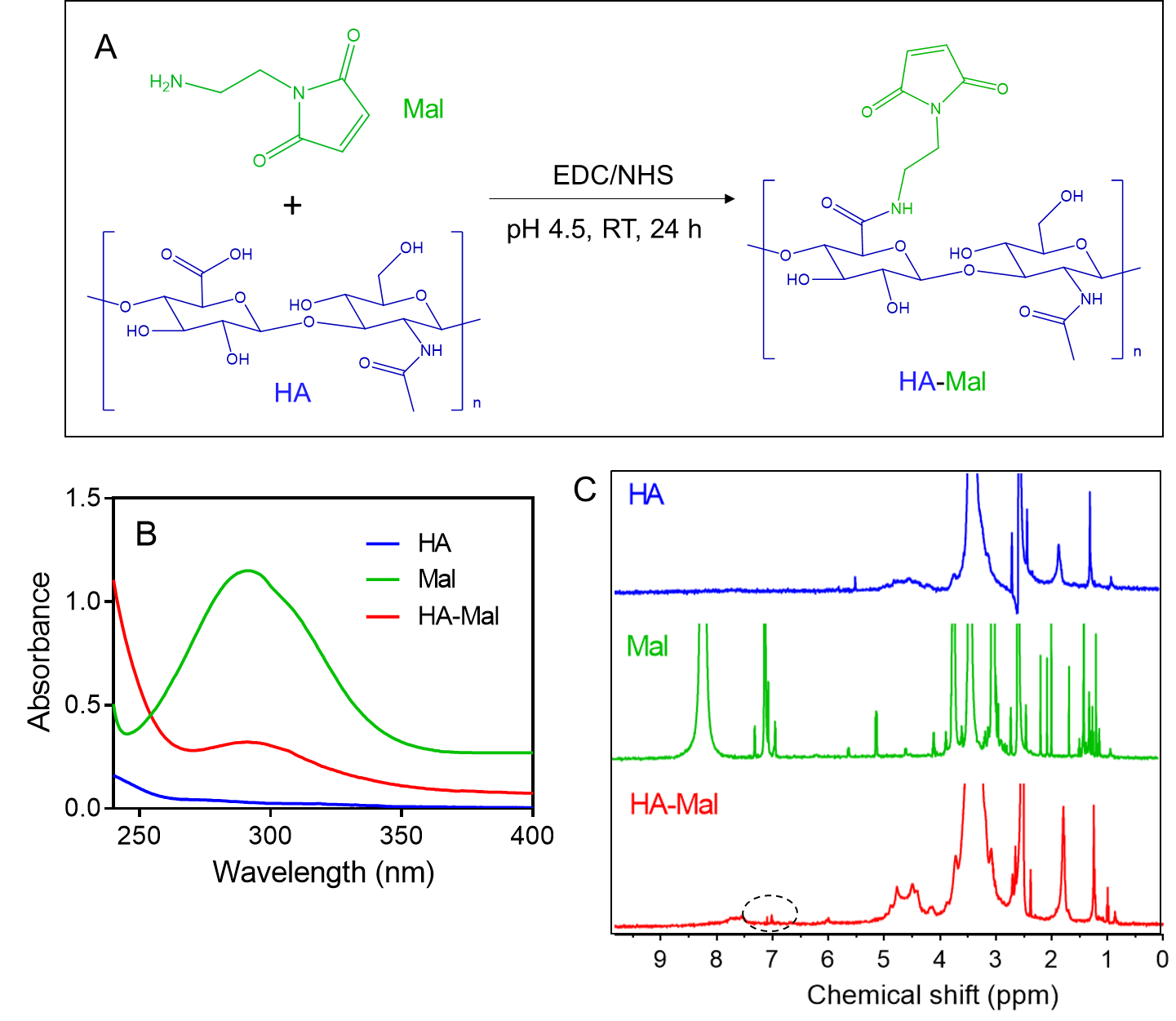


**Figure S6.** (A) A scheme showing the synthesis of HA-Mal. (B) The UV-Vis absorption spectrum and (C) ^1^H NMR spectrum of HA, Mal and HA-Mal. The characteristic UV-Vis absorbance (~280 nm) and proton shift (~7 ppm) corresponding to the vinyl protons of Mal were seen in HA-Mal, confirming the successful conjugation of Mal on HA.


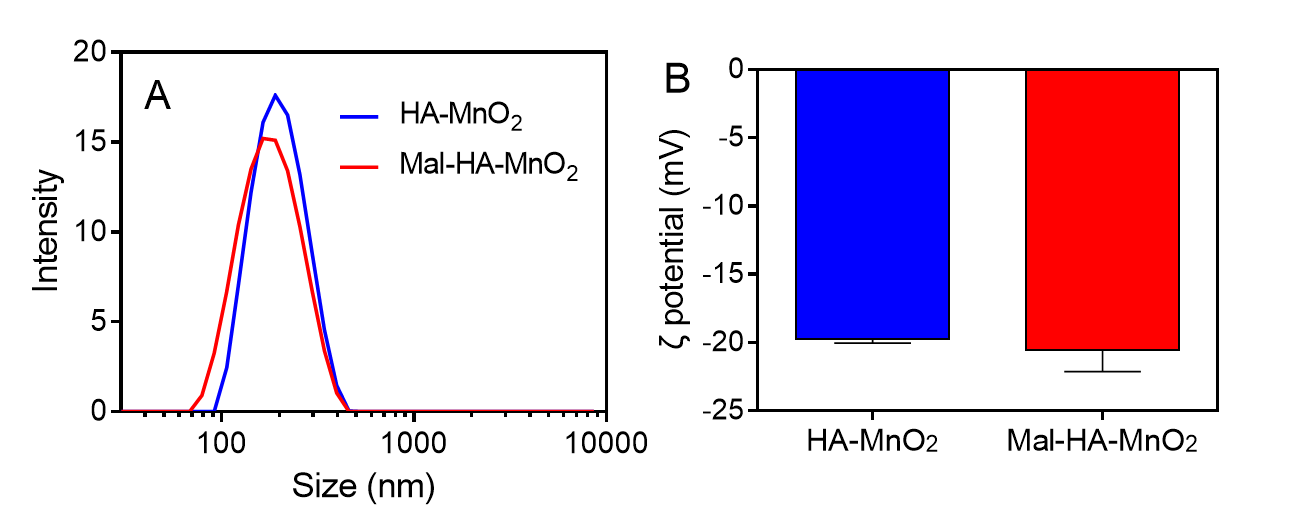


**Figure S7.** (A) The size distribution and (B) ζ potential of HA-MnO_2_ and Mal-HA-MnO_2_ (n=3). Data were presented as mean ± SD.

**Figure S8.** The Dz conjugation to HMN at different feeding concentrations determined by measuring the fluorescence intensity of the supernatant (n=3). Data were presented as mean ± SD.


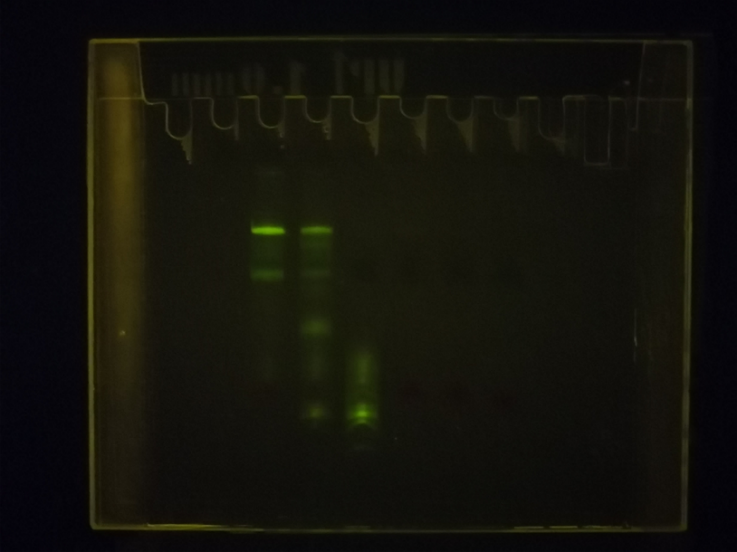


**Figure S9.** The original PAGE image for Figure 3F, which was cropped from this image post conversion to 8-bit and inversion by ImageJ software.


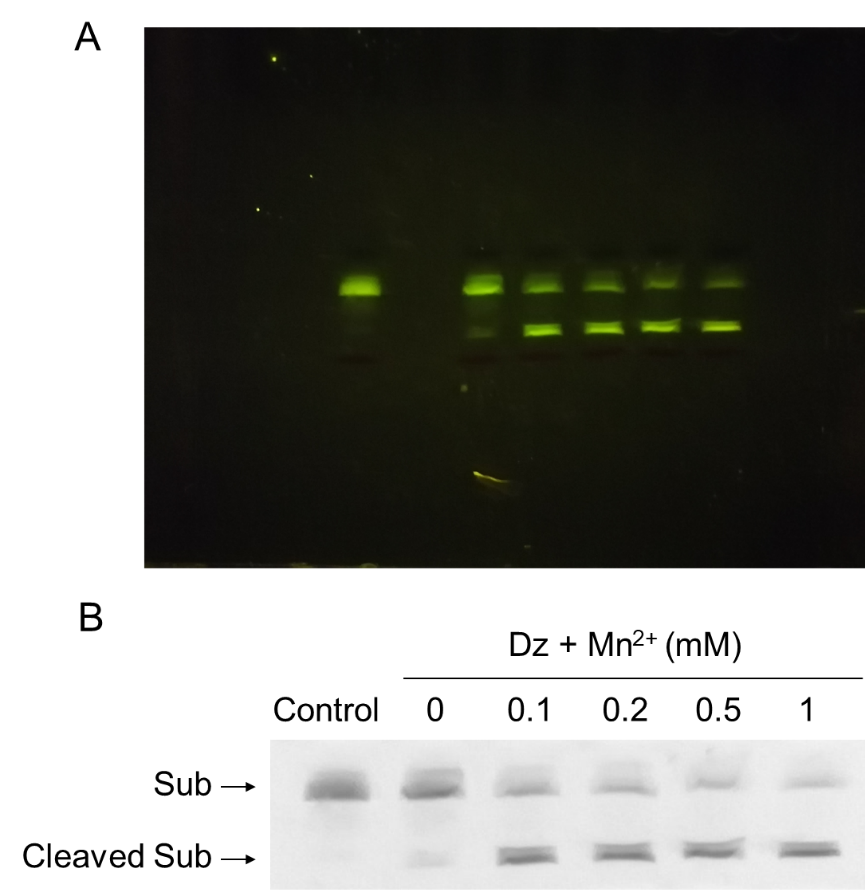


**Figure S10.** (A) The original PAGE image showing the cleavage activity of Dz in presence of different concentrations of Mn^2+^. (B) The image cropped from (A) post conversion to 8-bit and inversion by ImageJ software.


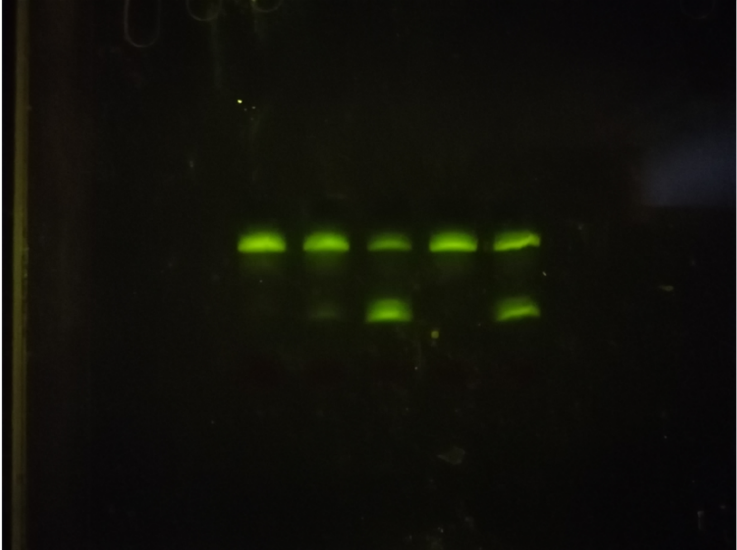


**Figure S11.** The original PAGE image for Figure 3H, which was cropped from this image post conversion to 8-bit and inversion by ImageJ software.


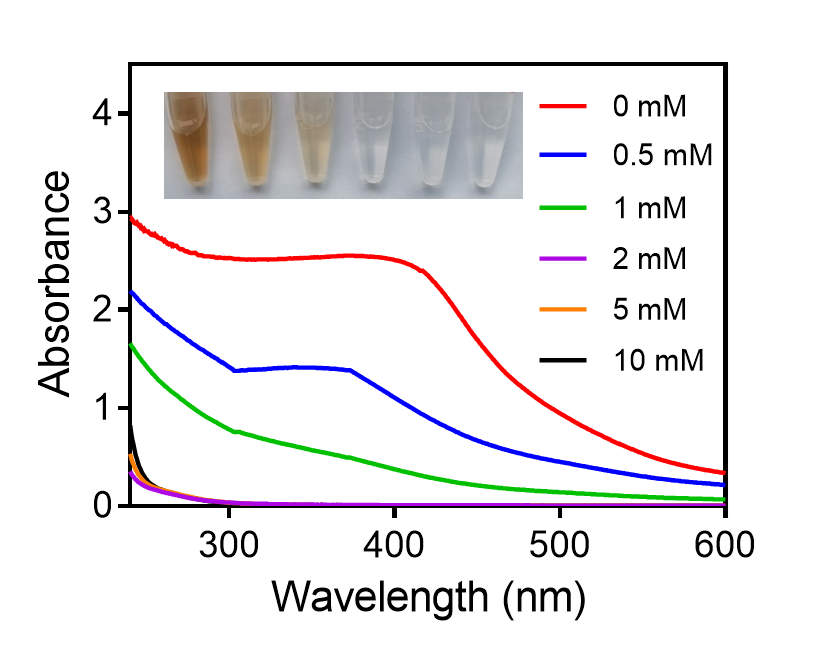


**Figure S12.** The UV-Vis absorption spectrum and appearance of DzMN in the presence of different concentrations of GSH. The absorbance of DzMN decreased accompanied by color fade as the concentration of GSH increased, suggesting the GSH-responsive degradation.

**Figure S13.** The viability of B16F10 cells after incubating with different concentrations of DzMN for 48 h (n=6). Data were presented as mean ± SD.


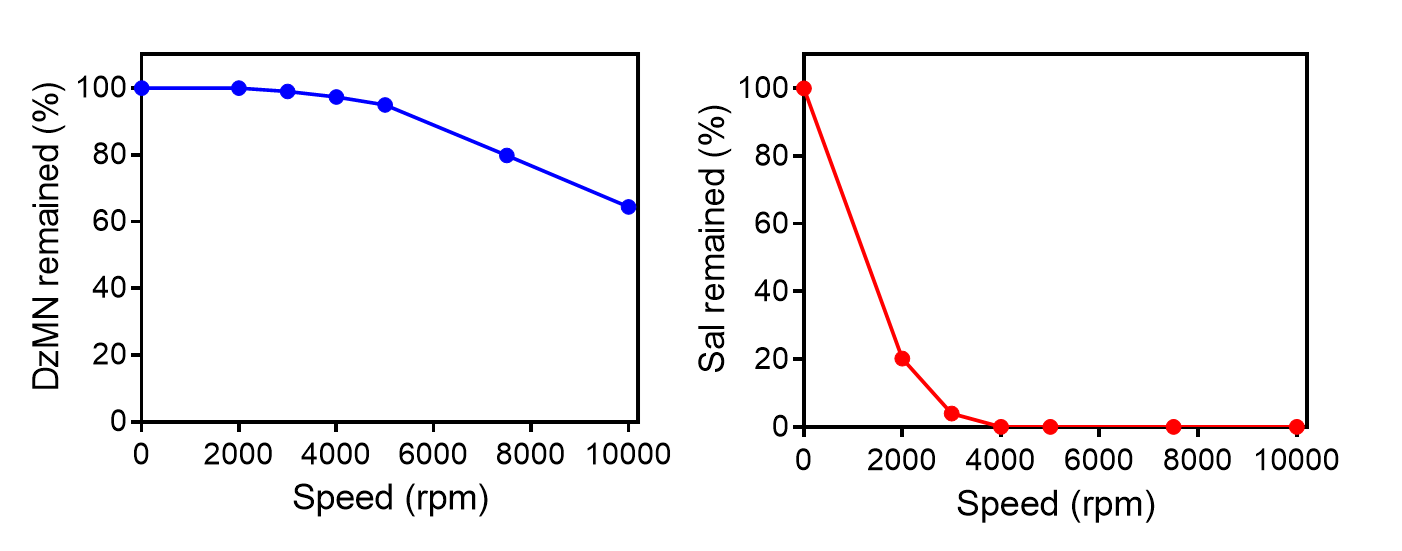


**Figure S14.** The optimization of centrifugation speed to precipitate *Sal*@PST/DzMN but not DzMN. The *Sal* and DzMN were centrifuged at various speed for 5 min, and the absorbance of the supernatant at 600 nm and 340 nm were measured to determine the remaining *Sal* or DzMN in the supernatant, respectively. Based on these results, the speed of 3000 rpm was chosen to separate DzMN from *Sal*@PST/DzMN.


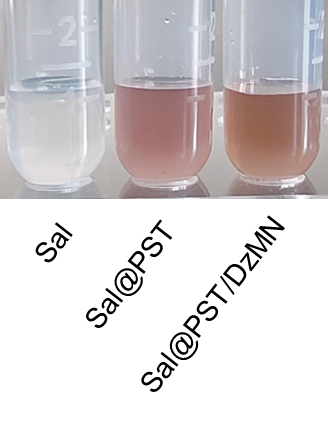


**Figure S15.** The photographs of *Sal*, *Sal*@PST and *Sal*@PST/DzMN.


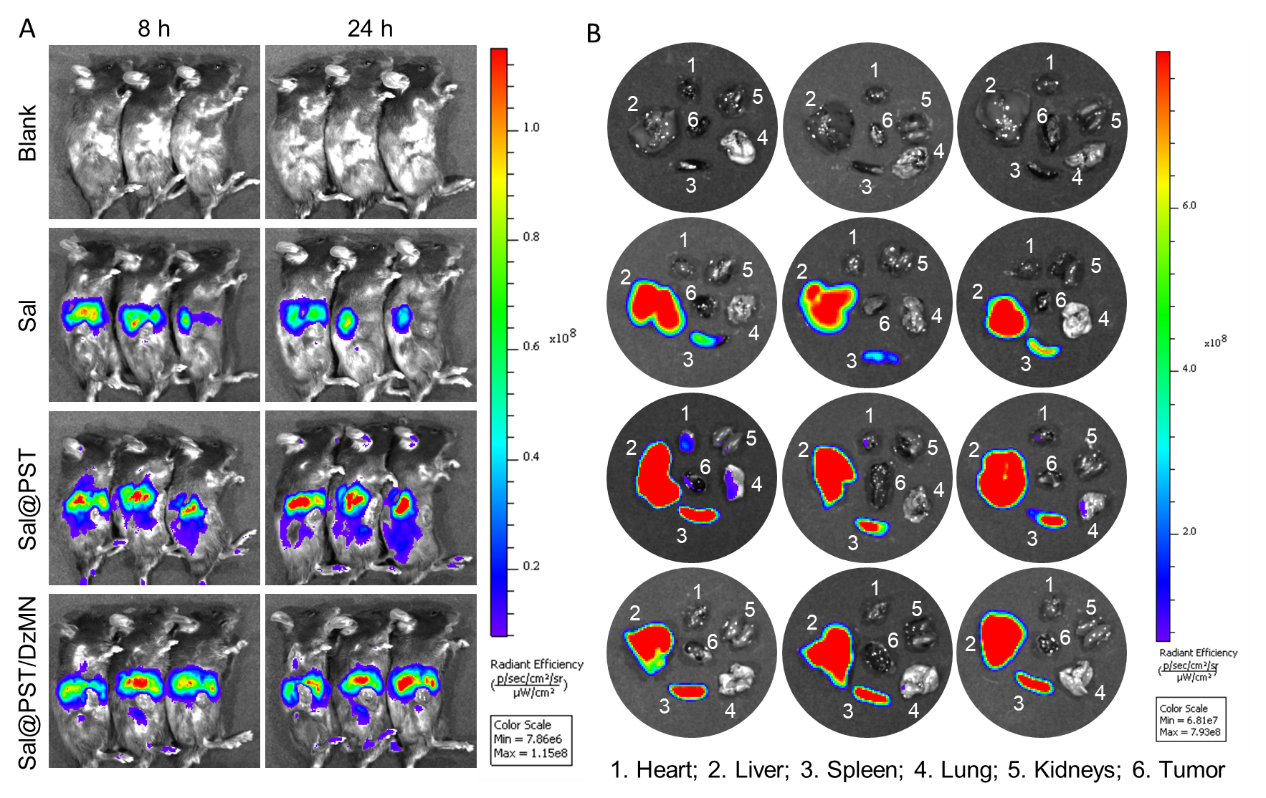


**Figure S16.** (A) *In vivo* and (B) *ex vivo* fluorescence imaging of mice post intravenous injection of DiR-labelled *Sal*, *Sal*@PST or *Sal*@PST/DzMN (n=3).


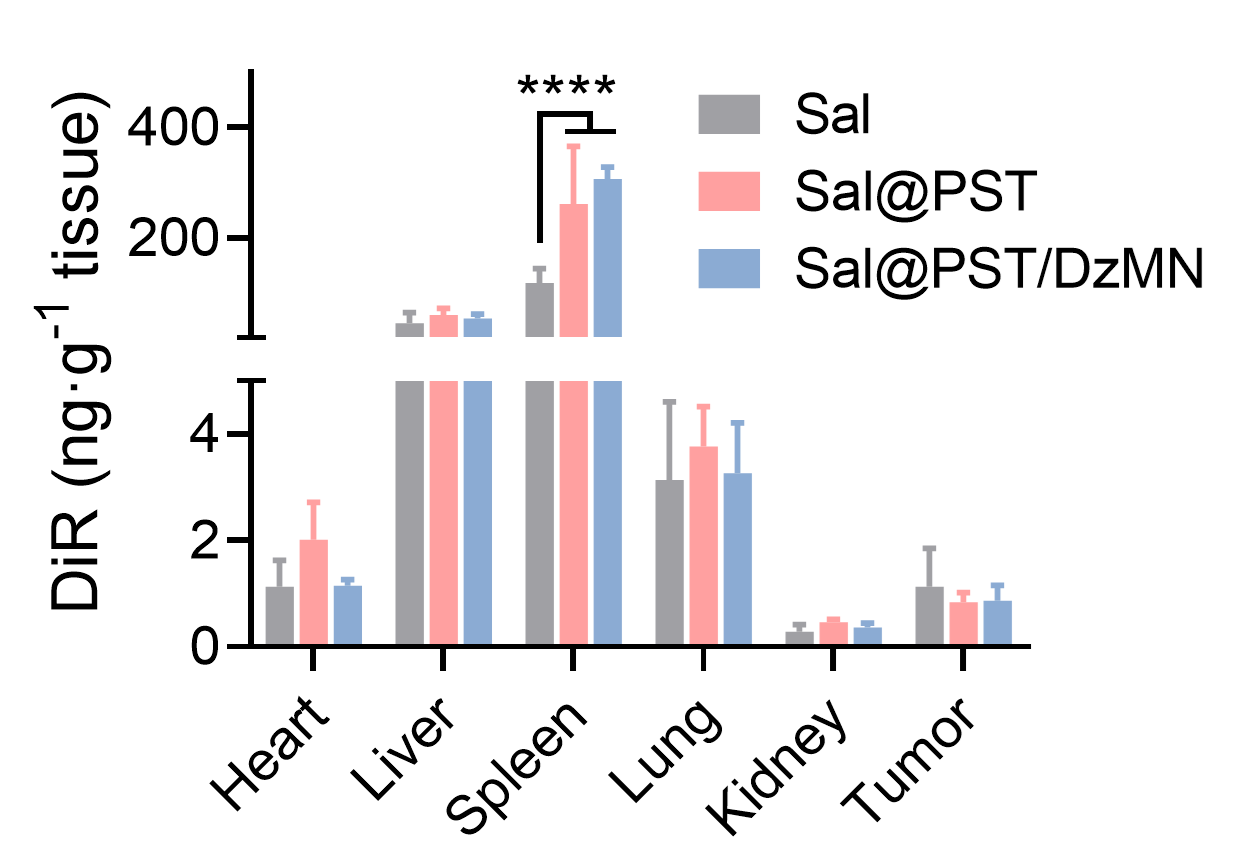


**Figure S17.** The DiR content in the tissues of mice post intravenous injection of DiR-labelled *Sal*, *Sal*@PST or *Sal*@PST/DzMN (n=3). Data were presented as mean ± SD. Statistical comparisons were performed using two-way ANOVA. *****p* < 0.0001.


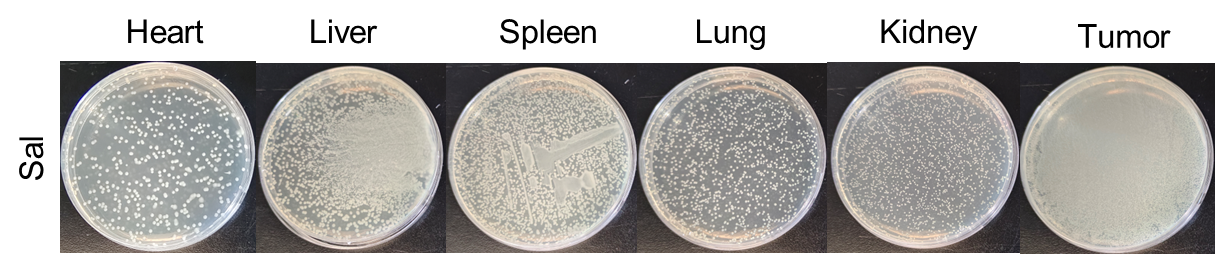


**Figure S18.** Representative plate photographs of tissues homogenates collected from mice at 24 h post *Sal* injection. Sporadic colonies grew from homogenates of major organs while dense colonies grew from tumor homogenates.


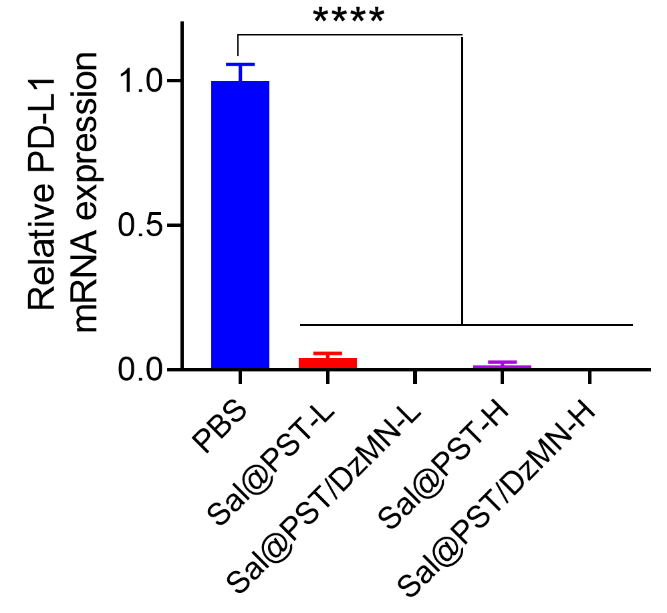


**Figure S19.** The relative mRNA expression of PD-L1 in tumor tissues of mice post different treatments (n=3). Data were presented as mean ± SD. Statistical comparisons were performed using one-way ANOVA. *****p* < 0.0001.

**References**

[1] K. M. Yoo, S. V. Murphy, A. Skardal, *Gels* **2021**, *7*, 13.
